# The Copper(II)-Assisted Connection between NGF and BDNF by Means of Nerve Growth Factor-Mimicking Short Peptides

**DOI:** 10.3390/cells8040301

**Published:** 2019-04-01

**Authors:** Irina Naletova, Cristina Satriano, Adriana Pietropaolo, Fiorenza Gianì, Giuseppe Pandini, Viviana Triaca, Giuseppina Amadoro, Valentina Latina, Pietro Calissano, Alessio Travaglia, Vincenzo Giuseppe Nicoletti, Diego La Mendola, Enrico Rizzarelli

**Affiliations:** 1 Department of Chemical Sciences, University of Catania, Viale A. Doria 6, 95125 Catania, Italy; irina_naletova@yahoo.com (I.N.); csatriano@unict.it (C.S.); alessio_travaglia@hotmail.it (A.T.); 2 Department of Health Sciences, University of Catanzaro, Campus Universitario Viale Europa, 88100 Catanzaro, Italy; apietropaolo@unicz.it; 3 Endocrinology, Department of Clinical and Experimental Medicine, Garibaldi-Nesima Medical Center, University of Catania, via Palermo n. 636, 95122 Catania, Italy; fiorenza.giani@gmail.com (F.G.); gpandini@gmail.com (G.P.); 4 Medicina Molecolare e Traslazionale “Rita Levi Montalcini”, Institute of Cellular Biology and Neurobiology (IBCN), National Research Council (CNR), c/o Policlinico Umberto I, University of Rome “La Sapienza”, Via del Policlinico 255, 00161 Rome, Italy; vtriaca75@gmail.com; 5 Institute of Translational Pharmacology (IFT), National Research Council (CNR), 00131 Rome, Italy; giusy.amadoro@gmail.com; 6 European Brain Research Institute, Viale Regina Elena 295, 00161, 64-65, 00143 Rome, Italy; valentina.latina@libero.it (V.L.); pietro.calissano@gmail.com (P.C.); 7 Section of Medical Biochemistry, Department of Biomedical and Biotechnological Sciences, University of Catania, 95124 Catania, Italy; 8 Department of Pharmacy, University of Pisa, Via Bonanno Pisano 6, 56126 Pisa, Italy; 9 Institute of Crystallography-Catania, National Research Council (CNR), Via P. Gaifami, 95126 Catania, Italy

**Keywords:** neurotrophin, metal ions, copper, TrK, p75, CREB, synapsin, Alzheimer’s disease, nerve growth factor, brain derived neurotrophic factor

## Abstract

Nerve growth factor (NGF) is a protein necessary for development and maintenance of the sympathetic and sensory nervous systems. We have previously shown that the NGF N-terminus peptide NGF(1-14) is sufficient to activate TrkA signaling pathways essential for neuronal survival and to induce an increase in brain-derived neurotrophic factor (BDNF) expression. Cu^2+^ ions played a critical role in the modulation of the biological activity of NGF(1-14). Using computational, spectroscopic, and biochemical techniques, here we report on the ability of a newly synthesized peptide named d-NGF(1-15), which is the dimeric form of NGF(1-14), to interact with TrkA. We found that d-NGF(1-15) interacts with the TrkA-D5 domain and induces the activation of its signaling pathways. Copper binding to d-NGF(1-15) stabilizes the secondary structure of the peptides, suggesting a strengthening of the noncovalent interactions that allow for the molecular recognition of D5 domain of TrkA and the activation of the signaling pathways. Intriguingly, the signaling cascade induced by the NGF peptides ultimately involves cAMP response element-binding protein (CREB) activation and an increase in BDNF protein level, in keeping with our previous result showing an increase of BDNF mRNA. All these promising connections can pave the way for developing interesting novel drugs for neurodegenerative diseases.

## 1. Introduction

The neurotrophins (NTs) are a structurally and functionally related family of growth factors that form non-covalently linked homodimers able to regulate cell survival, differentiation, neurite outgrowth and regeneration, and synaptic plasticity in both the central and the peripheral nervous system [1]. NTs include nerve growth factor (NGF), brain-derived neurotrophic factor (BDNF), neurotrophin-3 (NT-3), neurotrophin-4/5 (NT-4/5), neurotrophin-6, and neurotrophin-7 [2].

The biological functions of NTs are tuned through ligand-induced activation of two classes of cell surface receptors—the Trk receptors and the p75 neurotrophin receptor (p75NTR) [3]. 

The Trk receptors are tyrosine kinase receptors that dimerize upon NTs binding, resulting in autophosphorylation of intracellular tyrosine residues and signal transduction cascade [4]. Conversely, p75NTR is not a tyrosine kinase receptor but a member of the tumor necrosis factor receptor superfamily [5].

Among the NTs, the NGF is the first discovered cell growth factor and continues to raise a widespread interest [6]. NGF specifically acts through the binding to a tropomyosin receptors kinase A (TrkA) with high affinity or via a p75NTR receptor with low affinity. The TrkA receptor triggers pro-survival signaling, and p75 leads to activation of pro-apoptotic mechanisms [7].

The Trk receptor family shows high sequence homology and similar region organization in their extracellular portion. A leucine-rich region (domain 2) flanked by two cysteine-rich regions (domains 1 and 3) feature the first three domains. The fourth and fifth domains (Trk-d4 and Trk-d5) are immunoglobulin (Ig)-like domains, which are followed by a long linker connecting the extracellular portion of the receptor to the transmembrane region [8].

Neurotrophin binding to Trk receptors leads to activation or upregulation of downstream signaling cascades involved in neuron survival and synaptic plasticity; known pathways are phosphatidylinositol 3-kinase (PI3K)/protein kinase B (Akt), Ras/mitogen-activated protein kinase (Ras/MAPK), and phospholipase C (PLC) [9].

In this scenario, Trk receptors show three key recognition sites that modulate their activity for binding, activation, and regulation. The main activation site in each Trk corresponds to the D5 (IgC2) domain, the main regulatory site corresponds to the D4 (IgC1) domain, and the D1 (N-terminus) domain can act as an allosteric site depending on the presence of p75 [10,11].

Despite their high potential to be used as drugs in neurodegenerative diseases, the poor pharmacological profile of NTs, short half-lives, inability to penetrate the blood-brain barrier, undesirable high potency and pleiotropic effects, and the activation of multiple receptors has thwarted their use as therapeutic agents [12]. However, the knowledge of key functional domains of the Trk receptor allows developing agonists or antagonists [13,14].

A wealth of effort has been made through the years in combining simulation protocols with experimental research for enhancing a rational peptidomimetic design. Small molecules that mimic the NTs functionally or structurally have been designed and synthesized, expecting to be devoid of the aforementioned drawbacks [15,16,17,18,19].

Recently, small peptides encompassing the 1–14 sequence of the human NGF have been shown to activate NGF-TrkA receptor kinase cascade in PC12 cells with effects comparable with those NGF-induced [20,21]. In addition, NGF mimicking peptides triggered the phosphorylation of the transcription factor cAMP response element-binding protein (CREB), which represents a key transcriptional mediator of neuronal responses to neurotrophins [22]. The peptide-mediated phosphorylation of CREB resulted in increased expression of BDNF.

The NGF mimetic activity of NGF (1-14) peptides has been shown to be modulated by copper and zinc ions [21], although conflicting results have been reported on the role of these metal ions to regulate the NTs signaling [23]. Zinc and copper ions have been reported to have positive effects in several in vitro and in vivo models. Both metals rescued the level of NGF in a zinc(II) deficient mice [24], counteracted p75-driven apoptosis in chick neural retina [25], and blocked the binding of NGF to p75, attenuating its pro-apoptotic signaling cascade in chick embryonic cell cultures. 

Moreover, NGF is able to increase—up to 14-fold—the cellular level of copper in PC12 cells [26] and copper complexes with ionophore ligands promoting neurite elongation [27]. Other reports have shown that Zn^2+^ and Cu^2+^ (100 µM) inhibit the in vitro effects of NGF (as well as BDNF, NT-3, and NT-4/5), inducing conformational changes that alter the NGF binding to TrkA [28]. Metal ion binding to NGF has also been shown to: (i) block the NGF-mediated neurite outgrowth in chick dorsal root ganglia; (ii) decrease the cell viability [29]; and (iii) counteract the NGF-mediated protection from oxidative stress in PC12 cells [25,30].

**Scheme 1:**
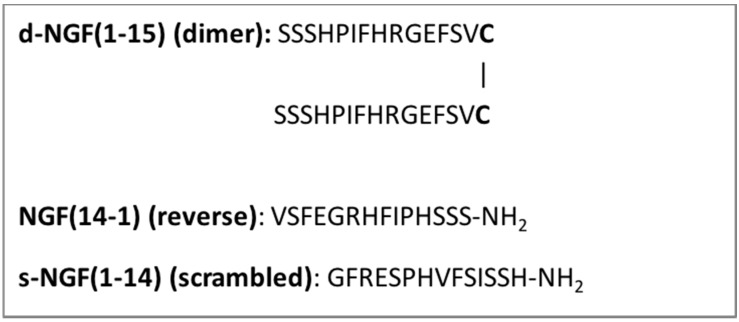
Amino acid sequence of dimer, reverse, and scrambled N-terminal nerve growth factor (NGF) peptides.

To further validate the NGF mimicking ability of these peptides from a structural and functional point of view, here we report on the ability of a newly synthesized peptide named d-NGF(1-15), which is the dimeric form of NGF(1–14) obtained via a cysteine-bridge linker of two monomeric units to interact with TrkA, and specifically with the D5 domain of TrkA (TrkA-D5), in comparison with the monomeric peptide NGF(1-14) encompassing the 1-14 amino acid sequence of NGF. As additional control, we used its reverse, NGF(14-1), or a scrambled peptide [hereafter named s-NGF(1-14)] (Scheme 1). Spectroscopic techniques were used to characterize the binding and the structural features of the copper(II) complexes with d-NGF(1-15), NGF(14-1), and s-NGF(1-14). Moreover, we scrutinized the different activation by these peptides of NGF-like signaling cascade in PC12 cells. Since these cells express low levels of TrkA [31], micromolar concentrations of mimetics were required, consistent with other literature reports for analogous NGF-mimetic peptides [32]. Furthermore, the different copper(II) ionophore capability of the peptides was studied, including the release of BDNF, and therefore the involvement of synapsin I and SNAP-25 proteins reported to act as downstream players of the brain activity of BDNF [33]. 

It is important to note that BDNF plays a critical role in neurobiological modifications related to pathophysiology of schizophrenia, depression, anxiety, and other psychiatric disorders [34,35,36].

Attempts have been made to use BDNF as therapeutic agent. Being a protein, the degradation of BDNF by proteolytic enzymes with a half-life widely reported to be far less than 30 min coupled with its inability to pass through the blood-brain barrier (BBB) is, however, the drawback of using it in therapy [37].

The potential metal assisted connection between NGF mimetics and BDNF could be relevant to increase the BDNF level in the brain [19].

Finally, we validated the previous hypothesized copper ability to inhibit tyrosine phosphatase activity [21]. 

## 2. Materials and Methods

### 2.1. Simulation Details

#### 2.1.1. Parallel Tempering Simulations

s-NGF(1-14), NGF(14-1), and d-NGF(1-15) peptides underwent 20 ns of parallel tempering (PT) simulations in explicit solvent with a total volume of 60 × 60 × 60 Å^3^ [100 × 70 × 70 Å^3^ for dimeric NGF(1-15)] after being equilibrated through 2 ns of Molecular Dynamics (MD) in explicit solvent. GROMACS 2016 package [38] was used. The AMBER99SB [39] force field was used for the biomolecules and counter ions, and the TIP3P [40] force field was used for water molecules. Electrostatic interactions were calculated using the Particle mesh Ewald method [38]. A cutoff (0.9 nm) was used for the Lennard-Jones interactions. The time-step was set to 2 fs. All bond lengths were constrained to their equilibrium values using the SHAKE [41] algorithm for water and the LINCS [42] algorithm for the peptide. We simulated 64 replicas distributed in the temperature range 300–400 K following a geometric progression. All replicas were simulated in NVT ensemble using a stochastic thermostat [43] with a coupling time of 0.1 ps. A thermostat that yields the correct energy fluctuations of the canonical ensemble is crucial in parallel tempering simulations [44]. Exchanges were attempted every 0.1 ps. The method of Daura and Van Gunsteren [45] was used in the post-processing phase to cluster the resulting trajectories with a cutoff of 3 Å calculated on the backbone atoms, as implemented in the clustering utility provided in the GROMACS package [42]. 

#### 2.1.2. Docking Simulations

The starting coordinates of domain-5 of TrkA (TrkA-D5) were taken from the X-ray structure of TrkA-D5 bound to NGF (pdb code 1WWW) [46]. The former complex was used as template for the alignment of the main MD clusters of s-NGF(1-14), NGF(14-1), and of the dimeric d-NGF(1-15) prior to the docking to TrkA-D5. Docking simulations were performed using HADDOCK interface [47]. All investigated peptides were included as active residues for the Haddock docking, as well as V288 to C300 belonging to TrkA-D5. Structures underwent rigid body energy minimization, semi rigid simulated annealing in torsion angle space, and a final clusterization of the results.

### 2.2. Peptide Synthesis

The NGF(1-14) peptide encompassing the amino acid sequence SSSHPIFHRGESFV-NH_2_, the scrambled s-NGF(1-14) GFRESPHVFSISSH-NH_2_, the reverse NGGF(14-1) VFSEGRHFIPHSSSNH_2_, and its dimeric form d-NGF(1-15) with the sequence SSSHPIFHRGESFV-C-C-VFSEGRHFIPHSSS was designed and synthesized with the C-termini amidated, as previously reported [48,49]. The fluorescent peptides were labeled with 5,6-carboxyfluorescein group through the side chain of an amidated additional lysine residue (purchased from CASLO, Lyngby, Denmark) to obtain NGF(1–14)Fam and d-NGF(1–15)Fam, respectively.

### 2.3. Spectroscopic Measurements

#### 2.3.1. UV-Visible Measurements

UV-Vis spectra were carried out on an Agilent 8453 spectrophotometer at 25 °C. The 200–800 nm range was explored, and quartz cuvettes with a 1 cm path length were employed. The concentration of the solutions containing peptides and copper(II) ion in a ratio 1:1 was 1 mM. 

#### 2.3.2. Circular Dichroism (CD) Measurements

Circular dichroism (CD) spectra were obtained in a constant nitrogen flow on a Jasco model 810 spectropolarimeter. The pH of aqueous solutions was adjusted by adding NaOH. The CD spectra of the Cu^2+^ complexes as a function of the pH were obtained in the λ = 200–400 and 300–800 nm wavelength regions. All solutions were freshly prepared with doubly distilled water. The Cu^2+^ and peptide concentrations used for acquisition of the CD spectra in the visible region were identical to those used in the UV-Vis measurements [50]. Far-UV CD spectra were acquired by using Cu^2+^ and peptide concentrations ranging from 5.0 × 10^−6^ to 1.0 × 10^−5^ mol dm^−3^. The spectra were recorded in the 190–280 and 280–750 nm range, as an average of 3 scans (scan rate 50 nm min^−1^; resolution 0.1 nm; path length 1 cm). 

### 2.4. Cell Experiments

#### 2.4.1. Materials

RPMI-1640 media and chemicals, unless otherwise stated, were obtained from Sigma-Aldrich (St. Louis, MO, USA). Streptomycin, L-glutamine, fetal bovine serum (FBS), and horse serum (HS) were obtained from Lonza (Basel, Switzerland). NGF was obtained from Invitrogen Laboratories (Paisley, U.K.). NGF was used at a concentration of 50 ng/mL (approximately 4 nM). The used antibodies are listed as follows. From Cell Signaling Technology (Danvers, MA, USA): anti-phospho-TrkA (Y490) (Cat # 9141, 1:1000 dilution); anti-TrkA (Cat # 2505, 1:1000 dilution); anti-phospho-Akt (S473) (Cat # 9271, 1:1000 dilution); anti-Akt (Cat # 4685, 1:1000 dilution); anti-phospho-Erk1/2 (T202/Y204) (Cat # 9106, 1:1000 dilution); anti-Erk1/2 (Cat # 9107, 1:1000 dilution); anti-phospho-CREB (S133) (Cat # 9191, 1:1000 dilution); anti-CREB (Cat # 9197, 1:1000 dilution) antibodies. Anti-GRB2 antibody (Cat # sc-17813, 1:500 dilution) was purchased from Santa Cruz Biotechnology (Santa Cruz, CA, USA). From Merck Millipore (Billerica, MA, USA): goat anti-mouse and anti-rabbit IgG secondary antibody horseradish peroxidase-conjugated (AP181P and AP307P, 1:3000 dilution); Anti-Synapsin I antibody rabbit AB1543P. From ThermoFisher (Waltham, MA, USA): Hoechst33342 and Halt Protease and Phosphatase Inhibitor Single-Use Cocktail. From Sigma-Aldrich: anti-SNAP-25 antibody (clone SMI 81) mouse 836,301 BioLegend; β-actin mouse S3062; anti-mouse IgG (whole molecule)-Peroxidase antibody A4416; anti-rabbit IgG (whole molecule)-Peroxidase antibody A9169.

#### 2.4.2. Rat Pheochromocytoma Cultures

Rat pheochromocytoma (PC12) cells were obtained from the American Type Culture Collection (Manassas, VA), cultured at passages between the 5 and 20 in RPMI-1640, and supplemented with 10% horse serum (HS), 5% fetal bovine serum (FBS), 2 mM l-glutamine, 50 IU/mL penicillin, and 50 µg/mL streptomycin. Cell cultures were grown in a humidified atmosphere of air/CO_2_ (95:5) at 37 °C in an incubator (Heraeus Hera Cell 150).

#### 2.4.3. Animals

Pregnant Wistar rats (Charles River Laboratories) at gestational age 17–18 (E17–18) were used for all the experiments of this study. 

Experimental protocols were carried out in accordance with the European Communities Council Directive of 22 September 2010 (2010/63/UE) regarding the care and use of animals for experimental procedures and with the Italian legislation on animal experimentation (Decreto L.vo 116/92).

#### 2.4.4. Septal Neurons Primary Cultures

Septal neurons were prepared from embryonic day 17/18 (E17/18), as previously described [51]. Chronic, 10–12 days in vitro treatment (D.I.V.), with NGF (100 ng/mL) under conditions of low supplementation (0.2%) with the culturing serum-substitute B27 selectively enriched the basal forebrain NGF-dependent cholinergic neurons (+36.36%) at the expense of other non-cholinergic populations, mainly GABAergic (−38.45%) and glutamatergic (−56.25%) [51]. Briefly, embryos were surgically removed, and septo-hippocampal areas were dissected from the cerebral tissue in ice-cold Hanks’ balanced salt solution (HBSS, Gibco, Life Technologies), freed of meninges, digested with 0.25% trypsin for 15 min at 37 °C, dissociated by trituration, and seeded as follows: 2 × 10^6^ cells on poly-l-lysine (SIGMA)-coated plates (BD Falcon, Durham, NC, USA; 353001) for biochemistry analyses and 1 × 10^5^ cells on glass coverslips in 24-well plates (BD Falcon; 351147) for immunofluorescence analyses.

The dissociated cells were plated in serum-free Neurobasal medium (Gibco, Life Technologies, Waltham, MA, USA) supplemented with B27 (Invitrogen Inc., Carlsbad, CA, USA) at a final 0.2% concentration in the presence of NGF (100 ng/mL) for 10–12 days. One day after plating, cytosine arabinoside (5 µM) was added to inhibit glial proliferation. Cultures were kept at 37 °C in a humidified incubator in a 5% CO_2_ atmosphere without further medium changes until used for experiments. 

#### 2.4.5. Proliferation Assay

PC12 cells were grown in RPMI medium supplemented with 1% HS, 0.5% FBS in 96-well plates at a density of 2 × 10^4^ cells per well were treated for 48 h with NGF peptides at concentrations of 50 µM with or without 1 µM Cu(II) or 50 µM 2,9-Dimethyl-4,7-diphenyl-1,10-phenanthroline disulphonic acid (BCS). Then, cells were stained with non-toxic specific vital stain for DNA Hoechst33342. After 15 min, fluorescence was analyzed by Varioscan spectrophotometer (ex 350 nm, em 461 nm). Results are represented as the increase in Hoechst33342 mean fluorescence with respect to controls and are means ± SEM of six replicas.

#### 2.4.6. Neurite Growth Assay

For the measurement of neurite growth, cells were seeded at a density 4 × 10^4^ cells per well in 300 µL complete medium on l-poly-lysinated 48 TPP^®^ tissue culture plates (Sigma-Aldrich) for 24 h. Cells were incubated with NGF protein or NGF peptides in RPMI medium supplemented with 1% HS and 0.5% FBS for 72 h. Afterwards, four fields per well were randomly examined under an inverted microscope. The cells were photographed and quantitative analysis of total neurite length was performed using the ImageJ software (v1.46d version, NIH).

#### 2.4.7. Protein Cellular Lysates Preparation

After treatment, PC12 cells were harvested with RIPA buffer (50 mM TRIS-HCl, pH 8.0, 150 mM NaCl, 0.5 mM EDTA, 1% Triton X-100, 0.5 mM EGTA, 1% NP40, 0.1% SDS) containing 0.5 mM EDTA, 1% Triton X-100, 0.5 mM EGTA and Halt Protease and Phosphatase Inhibitor Single-Use Cocktail. Cell lysates were incubated for 30 min and centrifuged at 14,000× *g* for 10 min. As to septal neurons primary cultures, total proteins were extracted by scraping the cells in ice-cold RIPA buffer plus 5% sodium deoxicholate plus proteases inhibitor cocktail (Sigma Aldrich, P8340) and phosphatase inhibitor cocktail (Sigma Aldrich, P5726/P2850) and centrifuged at 4 °C for 20 min at 13,000 rpm. The supernatant was then collected, and the amount of total protein was determined by Bradford assay (Protein Assay Dye Reagent Concentrate, BioRad, Hercules, CA, USA).

#### 2.4.8. SDS-PAGE, Western Blot Analysis and Densitometry

Equal amounts of protein were separated by SDS-PAGE in 4–12% Bis-Tris gels (Invitrogen) and transferred to nitrocellulose membranes (0.45 μm, GE healthcare, Little Chalfont, UK). The filters were blocked in TBS-T containing 5% non-fat dried milk for 1 h at room temperature or overnight at 4 °C. Proteins were visualized using appropriate primary antibodies. All primary antibodies were diluted in TBS-T and incubated with the nitrocellulose blot overnight at 4 °C. Incubation with secondary peroxidase coupled anti-mouse or anti-rabbit was performed by using the ECL system (Thermo Scientific SuperSignal West Pico, 34080; Amersham ECL Prime, RPN2232). The nitrocellulose membrane was then stripped with buffer Restore (Pierce, Rockford, IL) and subsequently re-probed with the specific antibodies for the unphosphorylated proteins. GRB2 was used as the loading control for all markers. The level of phosphorylation was calculated as the ratio between data from anti-phospho antibodies over those from the related not phosphorylated counterparts. Protein loading was monitored by normalization to β-actin and growth factor receptor-bound protein 2 (GRB2) [52]. Blots were scanned and quantitative densitometric analysis was performed by using ImageJ (http://imagej.nih.gov/ij/).

#### 2.4.9. Internalization of TrkA

To assay the TrkA receptor internalization, PC12 cells in RPMI medium supplemented with 0.1% BSA were stimulated for 30 min with 50 ng/mL NGF or 50 µM NGF peptides in the absence or presence of CuSO_4_ (1 µM) or BCS pre-treated medium (50 µM, 24 h), as previously described [21]. Briefly, the PC12 cells rinsed with cold phosphate buffered saline (PBS) were incubated with 1.5 mg/mL EZ-Link Sulfo-NHS-LC-Biotin (Pierce, Rockford, IL, USA) in biotinylation buffer (10 mM boric acid, 154 mM NaCl, 7.2 mM KCl, 1.8 mM CaCl_2_, pH 8.4) for 30 min. Cells were then rinsed twice with quenching buffer (192 mM glycine, 25 mM Tris-HCl, 1.8 mM CaCl_2_, 154 mM NaCl, pH 8.3) and lysed in lysis buffer (20 mM Tris-HCl, pH 7.5, 137 mM NaCl, 1% Nonidet P-40, 10% glycerol, 1 mM MgCl_2_, 1 mM EGTA, 1 mM Na_3_VO_4_, 20 mM β-glycerol phosphate, 20 mM NaF, 1 mM PMSF, and 1 mg/mL aprotinin and leupeptin). One mg of protein lysates were added to 100 μl of UltraLink Immobilized NeutrAvidin beads (Pierce, Rockford, IL). Samples were incubated with rocking at 4 °C for 2 h. NeutrAvidin beads were rinsed three times with lysis buffer, and 50 μl of Laemmli buffer was added to each sample. Samples were resolved by SDS-PAGE and immunoblotted for TrkA and P75^NTR^.

#### 2.4.10. Enzyme Linked Immuno Sorbent Assay (ELISA)

The medium after treatment with NGF peptides was collected at 24 h of incubation and diluted with the provided sample buffer (1:1) immediately prior to the assay. Levels of BDNF in PC12 cells supernatants were measured with Rat BDNF ELISA kit (ERBDNF, Thermofisher, Waltham, MA, USA).

#### 2.4.11. Confocal Microscopy Imaging

PC12 cells were seeded in glass bottom dishes (22 mm of diameter, WillCo-dish^®^, Willco Wells, B.V.) and pre-treated with l-poly-lysine at a density of 5 × 10^4^ cells per dish in complete RPMI medium for 24 h until cellular adhesion was attained. Immediately before the confocal microscope experiments, cells were rinsed with fresh RPMI complete medium without serum. Two treatment conditions were used to scrutinize the peptide uptake by PC12 cells and the peptide ionophore activity, respectively. 

For cellular uptake experiments, 50 μM NGF(1-14)-Fam or d-NGF(1-15)-Fam were added to Hoechst33342-stained live cells on the confocal microscope stage operating in x-y-t scan imaging mode. A total scan time of 40 min with scan intervals of 30 s was set; the acquisition parameters were kept constant for all the experiments. After the peptide addition, the solution was vigorously mixed to ensure a homogeneous peptide concentration in the whole liquid volume inside the Petri dish to avoid artefacts in the real time live cell imaging owing to different diffusion rates of the peptide molecules related to concentration gradients, as previously described [21]. For quantification of intracellular copper, the cells were treated for 15 min with 10 μM peptides in the absence or the presence of 1 μM CuSO_4_. After the incubation time, cells were stained with nuclear dye Hoechst33342 and selective and specific Cu(I) coppersensor-1 [53] (5 min, 1 μM), washed with phosphate buffer saline solution (10 mM PBS, 37 °C, pH = 7.4), and fixed with high purity 2% paraformaldehyde in PBS (pH = 7.4). 

Confocal imaging microscopy was performed with an Olympus FV1000 confocal laser scanning microscope (LSM), equipped with diode UV (405 nm, 50 mW), multiline Argon (457 nm, 488 nm, 515 nm, total 30 mW), HeNe(G) (543 nm, 1 mW), and HeNe(R) (633 nm, 1 mW) lasers. An oil immersion objective (60×O PLAPO) and spectral filtering system were used. The detector gain was fixed at a constant value, and images were taken for all the samples at random locations throughout the area of the well. The following channels were imaged in sequential mode to eliminate cross talk between the channels: blue (ex/em = 405/425–475 nm) for the emission of the Hoechst33342-stained nuclei; green (ex/em = 488/500–530 nm) for the Fam emission; red (ex/em = 543/560–700) for the BODIPY moiety of coppersensor-1 probe.

The image analysis was carried out using Huygens Essential software (by Scientific Volume Imaging B.V., The Netherlands). In futher detail, in the Huygens Object Analyzer, the primary and secondary surface pipes were set as the red channel of the copper sensor and the blue channel of Hoechst nuclear staining, respectively. Therefore, the cytoplasmic copper (total red emission intensity) was measured as the sum of all voxel values (“Sum”), while the nuclear copper (red emission intensity co-localizing with blue emission) was measured as voxels of the primary pipe intersecting with another object of the secondary pipe (“ColocV”), where a voxel is volume pixel, the smallest distinguishable box-shaped part of the image. 

### 2.5. Data Analysis

Data presented are representative of at least three independent experiments and are expressed as mean ± SEM. Statistical analysis of all the experiments in this study was performed by GraphPad Prism (GraphPadSoftware, San Diego, CA, USA), SPSS 17.0.0 for Windows (SPSS Inc., Chicago, IL, USA), using the appropriate test, one way Anova. Results were considered significant when *p* < 0.05. 

## 3. Results and Discussions

### 3.1. Computation Studies Reveal that NGF(1-14), but not NGF(14-1) nor sNGF(1-14) Interact with TrkA-D5 

The TrkA-D5 domain is the main driver of the interaction with NGF [54,55] and NGF-mimicking NGF(1-14) peptide [20]. The binding with NGF and related peptides can affect the TrkA conformation, thus decreasing the mobility of some receptors domains. Furthermore, peptides can cluster together and induce the dimerization trans-phosphorylation of Trk receptors.

To verify sequence specificity in the NGF mimicking of the short peptide containing the N-terminus of the neurotrophin, the molecular recognition of TrkA by the monomer NGF(1-14) was compared with that of the scrambled s-NGF(1-14) and the reverse NGF(14-1) peptide.

**Figure 1:**
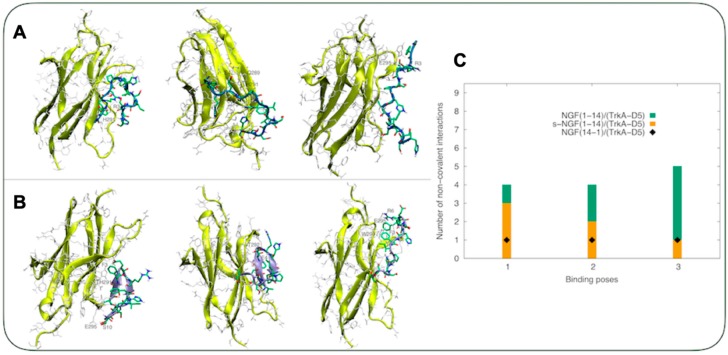
(**A**,**B**) From the top to bottom: the three lowest energy binding modes for s-NGF(1-14)/TrkA-D5 (**A**) and NGF(14-1)/TrkA-D5 (**B**) complexes, respectively. TrkA-D5 backbone is shown by yellow ribbons, each residue is shown by silver sticks. The s-NGF(1-14) and NGF(14-1) backbones are shown in blue and ice blue, respectively. Carbon atoms are shown in green, nitrogen atoms in blue, and oxygen atoms in red. (**C**) The number of non-covalent intermolecular interactions calculated for s-NGF(1-14)/TrkA-D5 and NGF(14-1)/TrkA-D5 compared with those calculated for the complex NGF(1-14)/TrkA-D5 [20].

The conformations of s-NGF(1-14) and NGF(14-1) obtained from parallel tempering simulations show marked differences compared to those of the wild type NGF(1-14) [21]. In particular, the short alpha helix spanning residues ^5^PIFHR^9^ was replaced by turn or random coil regions, as well as a beta sheet domain in the NGF(14-1) peptide. These conformational differences affected, in turn, the docking of the two peptides with domain 5 of TrkA (TrkA-D5) (Figure 1). 

In particular, upon s-NGF(1-14) and NGF(14-1) contact with TrkA-D5, three binding poses were detected for each peptide domain. A conserved region in TrkA-D5 was detected in the s-NGF(1-14)/TrkA-D5 complex, with similar residues found in the wild type complex. On the other hand, the binding site of TrkA-D5 to NGF(14-1) was located in a different region with respect to the one found in the wild type complex. 

Concerning s-NGF(1-14), the first binding pose involved a salt-bridge formed by Glu295 of TrkA-D5 and Arg3 of s-NGF(1-14). The second binding pose did not show any relevant weak non-covalent interaction apart from a CH-π contact formed by His291 of TrkA-D5 and His7 of s-NGF(1-14). The third binding pose was featured by a small network of hydrogen bonds involving Gln289 and His291 of TrkA-D5 and Glu4 of s-NGF(1-14). Moreover, His343 of TrkA-D5 faced Phe2 of s-NGF(1-14) (Figure 1A). The NGF(14-1) peptide bound to TrkA-D5 through Glu305 of TrkA-D5, which contacted Ser10 of NGF(14-1), while His301 of TrkA-D5 faced Phe3 of the peptide in the first binding pose. In a second cluster, Glu305 of TrkA-D5 contacted Arg6 of NGF(14-1), while Trp309 was observed to be perpendicularly oriented with respect to Phe3. In the third binding pose, non-specific interactions were detected despite a contact between Thr302 of TrkA-D5 and Glu4 of NGF(14-1) (Figure 1B). Overall, the number of non-covalent interactions was fewer compared to that found in the NGF(1-14)-TrkA-D5 complex (Figure 1C). Together, these findings reveal that only NGF(1-14), but not its reverse sequence NGF(14-1) nor the scrambled s-NGF(1-14) sequence, form strong and significant interactions with the domain D5 of TrkA receptor.

### 3.2. The Sequence-Dependent Interaction of Dimeric Peptide d-NGF(1-15) with Domain 5 of TrkA Is Greater than that of NGF(1-14)

**Figure 2:**
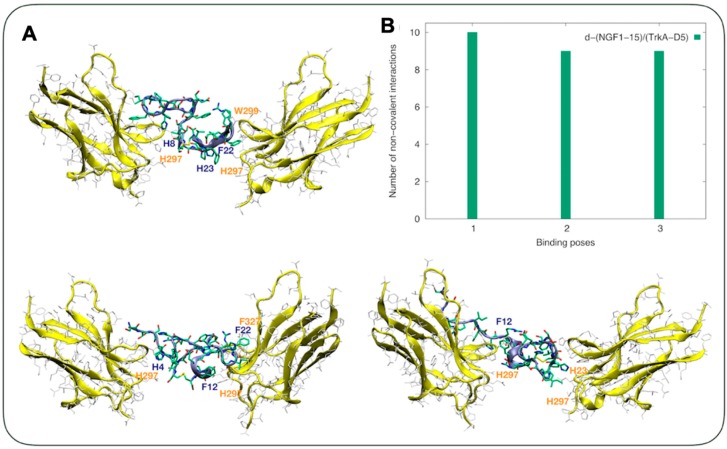
(**A**) The three lowest energy binding modes for d-NGF(1-15)/TrkA-D5. TrkA-D5 backbone is shown by yellow ribbons, each residue is shown by silver sticks. The d-NGF(1-15) residues are shown by sticks. Carbon atoms are shown in green, nitrogen atoms in blue, and oxygen atoms in red. (**B**) The number of non-covalent intermolecular interactions calculated for the d-(NGF1-15)/TrkA-D5 complex.

The secreted mature neurotrophins are soluble proteins that exist as non-covalently linked homodimers [56], thus we extended the computational study to the dimeric NGF peptide d-NGF(1-15). The structure of d-NGF(1-15) dimeric form obtained from molecular simulations showed flexible domains with segments of alpha helixes that easily converted to beta turns spaced by short sections of beta sheets (Figure 2).

In particular, beta turn regions were adopted along the dimeric domain (Figure 2A) as well as in the ^4^HPIFH^8^, where a short alpha helix was present in the X-ray structure (pdb code 1WWW). Short alpha helix domains were present in the regions ^10^GEFS^13^ and ^25^EFS^27^ (Figure 2A). Upon d-NGF(1-15) contacting the TrkA-D5 domain, three main binding poses were detected. All of them showed mainly aromatic interactions between the dimeric peptide and the TrkA receptor. In the first binding pose, H23 and F22 belonging to d-NGF(1-15) faced H297 and W299 of the TrkA receptor, respectively. In the second binding pose, F12 belonging to d-NGF(1-15) faced H297 of the TrKA receptor. F12 belonging to the d-NGF(1-15) faced H297 and H298 of the TrkA receptor in the third binding pose as well, where a salt bridge involving R24 of d-NGF(1-15) and E331 of TrkA-D5 was also detected. The former weak non-covalent interactions were detected in higher quantity compared with the complex formed by NGF1-14 and TrkA-D5 [20] and considering the number normalized per complex unit (Figure 2B). 

Overall, shallow interactions between TrkA and the NGF mimetic peptides were evident in the present study, in agreement with what previously suggested [57]. s-NGF(1-14) and NGF(14-1) peptides also interacted as monomers with one TrkA-D5 chain (Figure 1A), although their non-covalent interactions were significantly fewer than those formed by the wild type NGF(1-14) (Figure 1C). Indeed, the lower number of non-covalent interactions calculated for the scramble and reverse NGF1-14 peptides stressed that the recognition binding domain was sequence-dependent. Moreover, the docking simulations showed a directionality of binding. Reverting the sequence had an impact on the conformational ensemble of the NGF14-1 reverse peptide in comparison to the NGF1-14 wild-type analogue (Figure 1C). Beta-sheet domains tended to be adopted at variance of the central alpha helix typical of the wild-type peptide. A wild-type sequence was therefore required in order to allow the specific interactions typical of NGF binding to TrkA. This profound impact of the non-covalent interactions in the binding between NGF and TrkA-D5 was also straightforwardly shown from the binding of dimeric NGF1-15 with TrkA-D5. In particular, the NGF dimer bound through a disulfide bridge to both TrkA-D5 chains, and it featured a very similar orientation to the one adopted in the heterodimer structure of NGF/TrkA-D5 [46]. Here, all the binding poses contacted the TrkA-D5 section via non-covalent aromatic interactions mainly through His4, His8, F12, H23, or F22. Intriguingly, the disulphide bridge did not interact with any of the two TrkA-D5 chains, acting only as a link between the two NGF N-terminal domains. This connection of the disulfide bridge allowed it to keep a very similar orientation with the one of the full NGF peptide in the heterodimer complex with TrkA-D5 [46].

### 3.3. Cu^2+^ Interaction with d-NGF(1-15) Is Similar and Slightly Stronger than that with NGF(1-14), but Significantly Different Compared with that With s-NGF(1-14) and NGF(14-1)

It has been previously shown that NGF and related peptides can bind copper and zinc ions [21,48,58], and that these interactions can significantly modulate their biological activity. Here, UV-Vis and CD spectroscopic studies were run to characterize the complexes of the copper(II) with the d-NGF(1-15), sNGF(1-14), and NGF(14-1) and compare them with those of NGF(1-14). At physiological pH, the main species formed by d-NGF(1-15), s-NGF(1-14), and NGF(14-1) displayed UV-vis parameters (Table 1) indicative of the presence of three nitrogen donor atoms (NH_2_, N^-^_amide_, N_Im_) involved in a metal pseudooctahedral distorted coordination geometry [59,60], similar to what was observed for the NGF(1-14) peptide [48]. 

The CD spectra confirmed the coordination environment involving NH_2_, N^-^_amide_, and N_Im_ nitrogen atoms (Table 1 and Figure S1). Specifically, the bands at the wavelengths of about 282-288 nm, 310-328 nm, and 330–340 nm were assigned to NH_2_→Cu^2+^, N^-^_amide_→Cu^2+^, and N_Imidazole_ →Cu^2+^ π_1_ charge transfers, respectively [61,62,63]. The CD band at the wavelength of 550–632 nm was characteristic of the copper d-d- transition [61,62]. The spectrum of d-NGF(1-15):Cu displayed a 15 nm blue shift in λ_max_ (corresponding to the maximum absorbance) in comparison to NGF(1-14):Cu, which was indicative of the presence of a stronger field around the metal, likely due to the presence of an extra imidazole nitrogen in metal coordination environment of d-NGF(1-15) [62]. As to the s-NGF(1-14) and NGF(14-1) peptides, both complexes showed a slight red-shift in the UV-Vis λ_max_ absorbance band with a lower molar absorptivity coefficient, which suggested a lower equatorial field with a planar disposition of donor atoms [61].

Copper(II) and zinc(II) ions have been reported to affect the conformation and receptor binding, inhibiting the biological activities of NGF, BDNF, and NT-3 [28,30,64]. Far-UV CD allows for the determination of the effect of metal ion on the conformation of the d-NGF(1-15) peptide (Figure S2) in comparison with the monomer previously investigated [48]. The secondary structure of d-NGF(1-15) indisputably changes upon Cu^2+^ addition, as already reported for NGF(1-14). The CD spectra indicated a reduced random coil of the NGF N-terminus domain and an increase in the turn conformation, resulting from the deprotonation of the amide nitrogen atoms by Cu^2+^ coordinated to the imidazole nitrogen atoms of His4 and His8 (Figure S2a). The metal ion interaction with some of the residues involved in the interaction with the D5 domain of the receptor, as put in evidence by in silico studies (see above), gave rise to a more structured peptide, suggesting a favorable contribution of Cu^2+^ to the binding with the receptor.

The conformation of NGF(14-1) and s-NGF(1-14) was random coil, and the addition of copper led to a variation of the secondary structure with the appearance of a maximum centered around 220 nm (Figure S2b,c). This effect was greater in the copper complex formed by s-NGF(1-14), in agreement with a greater distortion of the coordination environment detected by the UV-Vis parameters. 

### 3.4. d-NGF(1-15), NGF(1-14), and NGF Affect Proliferation and Morphology/Differentiation of PC12 Cells Conversely s-NGF(1-14) and NGF(14-1) Do Not

**Figure 3:**
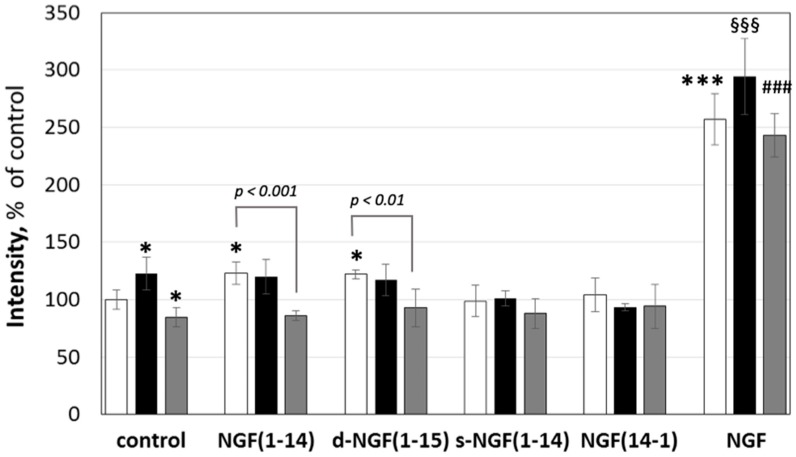
Effect of nerve growth factor (NGF) or NGF(1-14) peptides on the viability of PC12 cells. Quantification of number of cells by fluorescence emission of Hoechst33342-stained nuclei (ex/em = 350/461 nm). PC12 cells were grown with NGF (50 ng/mL) or NGF peptides (50 μM) in the absence (☐) or in the presence of 1 μM CuSO_4_ (■) or 50 μM BCS (■) for 48 h. Data are the mean ± SEM of four independent experiments, (* *p* < 0.05 and *** *p* < 0.001 versus control; §§§ *p* < 0.001 versus CuSO_4_; ### *p* < 0.001 versus BCS; one-way Anova).

To study the trophic effect of NGF peptides, we verified the effect of d-NGF(1-15) in comparison with s-NGF(1-14), NGF(14-1), NGF(1-14), and whole NGF protein in the presence or in the absence of 1 µM copper. Copper ions normally present in the culture medium at μM level [65] affect the proliferative effect of the NGF peptides [21]. Therefore, we treated cells with or without 50 µM of membrane-impermeable copper chelator BCS [66] (Figure 3).

The copper addition to the culture medium significantly increased the number of cells (123 ± 12% of untreated control), whereas the BCS treatment decreased it (85 ± 7%), thus demonstrating that the copper present in the culture medium contributes to the proliferation at the basal level. NGF(1-14) and d-NGF(1-15) alone showed a proliferative effect (123 ± 8% and 122 ± 3%, respectively); on the contrary, s-NGF(1-14) and NGF(14-1) did not induce any statistically significant increase in the cell number. It is noteworthy that the addition of copper ions did not increase the proliferative effect of free peptides. Conversely, BCS induced a significant decrease of cell number due to by NGF(1-14) (86 ± 3%). As expected, NGF treatment largely increased PC12 cells proliferation (257 ± 19%), which was further favored by the addition of copper (294 ± 29%), while BCS addition caused a decrease in cell proliferation (243 ± 16%). Overall, the effect of BCS was modulated by the different affinity of both the NGF and the peptides for copper(II) ion.

**Figure 4:**
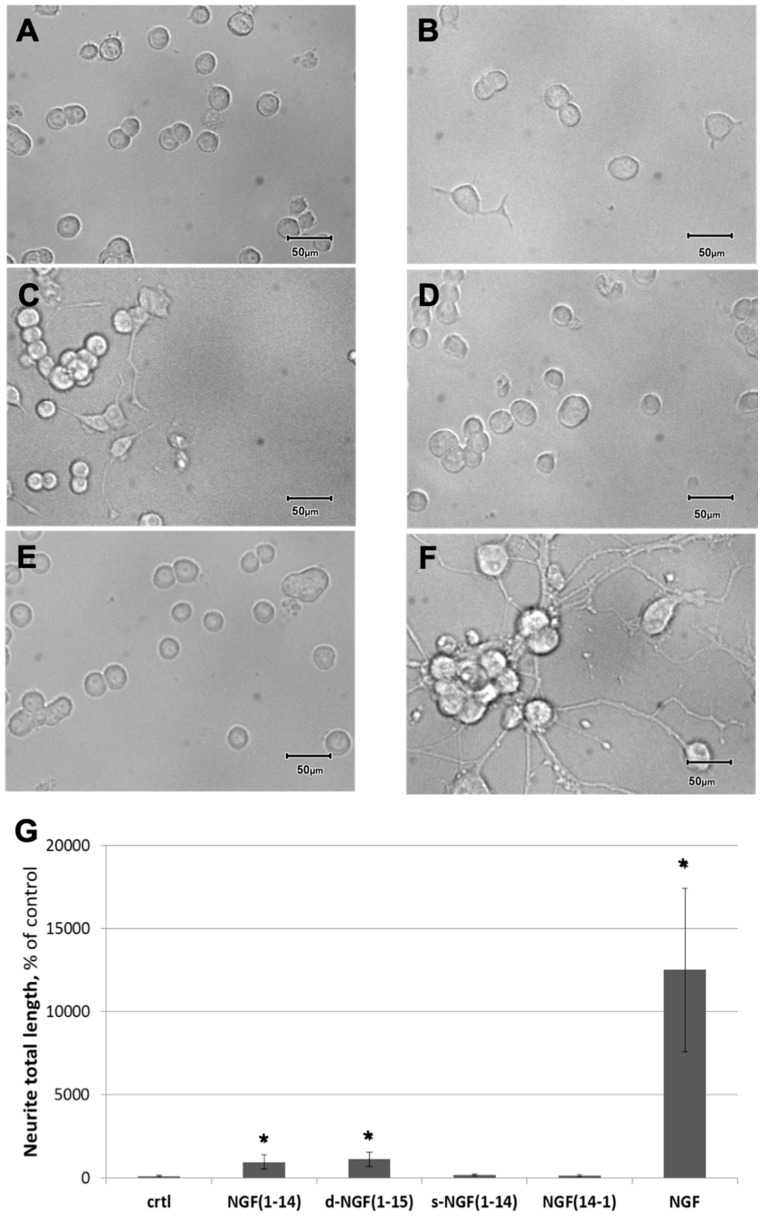
Effect of NGF or NGF(1-14) peptides on neuronal phenotype differentiation. (**A**–**F**): Photomicrographs obtained by microscopy of PC12 cells treated with 50 ng/mL NGF or 50 μM NGF(1-14) peptides for 72 h. Representative photos obtained from at least three independent experiments for: (**A**) unstimulated control, (**B**) NGF(1-14), (**C**) d-NGF(1-15), (**D**) s-NGF(1-14), (**E**) NGF(14-1), (**F**) NGF. Scale bar = 50 μm. (**G**): Percentage of neurite total length of PC12 cells treated with 50 ng/mL NGF or 50 μM NGF(1-14) peptides for 72 h, (* *p* < 0.05 versus control, one-way Anova).

Since the neurite outgrowth is a macro marker of neuronal differentiation, we treated cells (on l-poly-lysinated multiwell) for 72 h with or without protein (NGF, 50 ng/mL) or the 50 µM of NGF peptides (Figure 4).

As expected, NGF triggered the PC12 differentiation, as displayed by the formation of a complex neuronal network (Figure 4F). NGF(1-14) (Figure 4B) or d-NGF(1-15) (Figure 4C) induced a slight neurite outgrowth. Conversely, neither s-NGF(1-14) (Figure 4D) nor NGF(14-1) (Figure 4E) induced any differentiation of PC12 cells. Figure 4G shows the effect of protein (NGF, 50 ng/mL) or the 50 µM of NGF peptides on the total length of neurite.

Treatment with the whole protein or NGF(1-14) or d-NGF showed a significant increase of neurite outgrowth with 12,507 ± 4905%, 950 ± 424%, and 1118 ± 433% for the whole protein and peptides, respectively. It is important to note that incubation with s-NGF(1-14) or NGF(14-1) did not show a change in the neurite length.

### 3.5. The d-NGF(1-15) Is a Better TrkA (Y490) Activator than NGF(1-14), while s-NGF(1-14) and NGF(14-1) Do Not Induce Any Significant Effect

**Figure 5:**
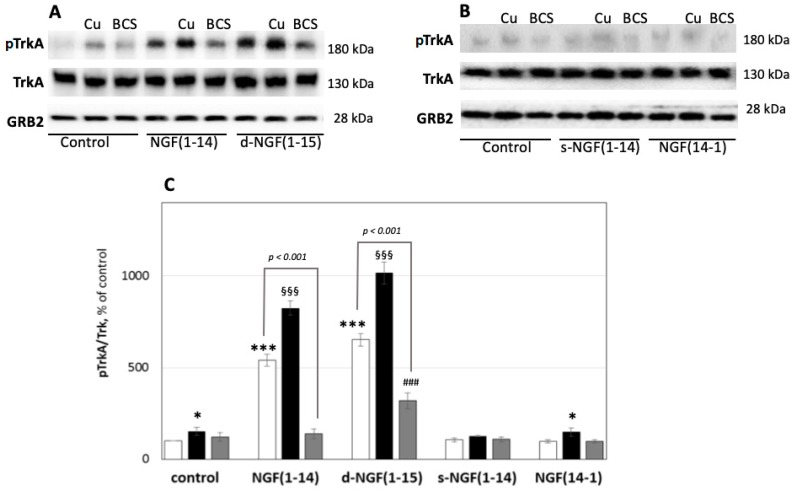
Effect of NGF or NGF(1-14) peptides on TrkA phosphorylation. Representative western blotting (**A**,**B**) and densitometric analyses (**C**) of pTrkA in PC12 cells treated with 50 μM NGF peptides in the absence (☐) or in the presence of 1 μM CuSO_4_ (■) or 50 μM BCS 24 h pre-treated medium (■) for 10 min. Data obtained from at least three independent experiments. The phosphorylated level of TrkA was reported as the ratio over the corresponding total protein (* *p* < 0.05, *** *p* < 0.001 versus control; §§§ *p* < 0.001 versus CuSO_4_; ### *p* < 0.001 versus BCS; one-way Anova).

The first event upon NGF binding to TrkA is the receptor phosphorylation at tyrosine 490 [67]. To investigate the ability of d-NGF(1-15) and NGF(1-14), we stimulated the PC12 cells with 50 μM NGF peptides for 10 min (Figure 5). 

The d-NGF(1-15) induced phosphorylation of TrkA (653 ± 34%) of the untreated control significantly more than the NGF(1-14) (540 ± 32%), while the addition of CuSO_4_ further increased Tyr-490 phosphorylation of TrkA (1017 ± 59%), a trend similar to that shown by the monomer NGF(1-14) in the presence of copper (825 ± 38%). The cell treatment by s-NGF(1-14) or the NGF(14-1) did not affect the TrkA phosphorylation, whereas the addition of CuSO_4_ increased NGF receptor phosphorylation for both s-NGF(1-14) and NGF(14-1); this effect appeared similar to that shown by copper(II) ion addition alone. Pre-treatment of PC12 cells with BCS at 50 µM for 24 h decreased TrkA phosphorylation induced by d-NGF(1-15) and NGF(1-14) (320 ± 43% and 138 ± 33%, respectively). 

In agreement with the in silico results, these experimental findings not only reconfirm the effect of NGF(1-14) but also give evidence of the stronger effect of d-NGF(1-15) in activating TrkA. Furthermore, the comparison between the receptor phosphorylation ability of monomeric and dimeric peptides containing the N-terminus of the protein with that shown by s-NGF(1-14) and NGF(14-1) indicates the relevant role played by the wild-type sequence. Copper(II) ion is involved in the phosphorylation process and potentiates the activity of the NGF mimicking activities of NGF(1-14) and d-NGF(1-15) differently. It is reasonable to state that the major gain of the dimer in comparison to the monomer in the activation of TrkA is due to its stronger ability to bind the metal ion, as suggested by our spectroscopic data. This hypothesis was further confirmed by results obtained with the contemporary addition of BCS with peptides—BCS reduced NGF(1-14) activity by 75%, whereas d-NGF(1-15) was less affected (55%).

### 3.6. d-NGF(1-15) Induces TrkA Receptor Internalization in PC12 Cells only in the Presence of CuSO_4_ Whereas Does Not Affect p75 Receptor Internalization

**Figure 6:**
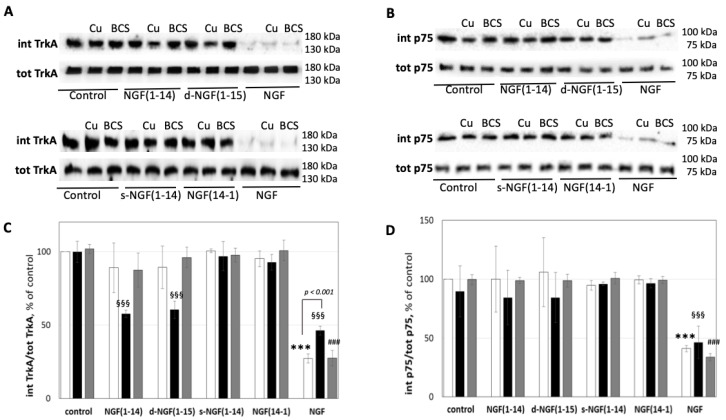
Effect of NGF(1-14) peptides on internalization of TrkA and p75 receptors. Representative western blotting (**A**,**B**) and densitometric analyses (**C**,**D**) of TrkA and p75 in PC12 cells treated with 50 μM NGF peptides in the absence (☐) or in the presence of 1 μM CuSO_4_ (■) or in 50 μM BCS 24 h pre-treated medium (■) for 30 min. Data obtained from at least three independent experiments. Crude cellular lysates run in parallel for total level of TrkA and p75 (*** *p* < 0.001 versus control; §§§ *p* < 0.001 versus CuSO_4_; ### *p* < 0.001 versus BCS; one-way Anova).

Receptors internalization is fundamental in the intracellular signaling, and BCS was used instead of blocking agents of TrkA internalization to put into evidence the potential signaling role of copper ions. In Figure 6, the ratio between TrkA still present in the membrane and the total amount is reported. Then, the internalization of the receptor was determined as a decrease in the western blot and quantified as the difference with the relative control. Both d-NGF(1-15) and NGF(1-14) induced a similar TrkA receptor internalization (60 ± 6% and 58 ± 3%, respectively, compared to the relative untreated control) only in the presence of CuSO_4_ (1 μM) (Figure 6). 

As expected, NGF alone elicited TrkA receptor internalization (72 ± 5%); the process was slightly inhibited by the addition of copper (54 ± 3%) and almost unchanged in the presence of BCS (73 ± 5%) (Figure 6A,B). The p75 internalization paralleled the TrkA pattern for NGF, while no statistically significant differences with respect to untreated cells were found for the treatments with all the NGF peptides (Figure 6B,C). CuSO_4_ induced a different behavior between the wild type NGF peptide fragments and the scrambled and reverse peptides studied herein. Intriguingly, contrary to the NGF protein, the interaction of NGF(1-14) and d-NGF(1-15) peptides with metal ion favored TrkA receptor internalization in a p75^NTR^–independent mode.

### 3.7. d-NGF(1–15) Signaling via TrkA Leads to Activation of cAMP Response Element-Binding Protein

The binding of NGF to its TrkA receptor triggered many cellular signaling responses that occurred via different pathways [3,8], including two major tyrosine kinase-mediated pathways—the phosphoinositide 3-kinase (PI3K)-AKT (also known as protein kinase B, PKB) [68] and the mitogen-activated protein kinase (MAPK)-extracellular signal-regulated kinase (ERK) pathway [69]. All pathways converged to signal to the cAMP-response element-binding protein (CREB), a transcription factor which binds to the promoter regions of many genes associated with synapse re-modeling, synaptic plasticity, and memory [70,71].

**Figure 7:**
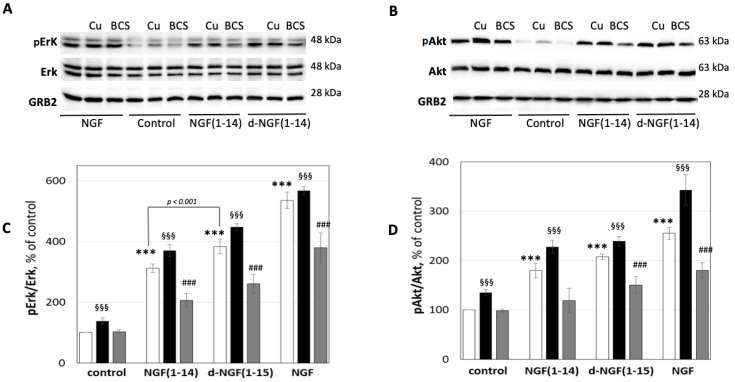
Effect of NGF or NGF(1-14) peptides on Erk1/2 and Akt phosphorylation. Representative western blotting (**A**,**C**) and densitometric analyses (**B**,**D**) of pErk1/2 and pAkt in PC12 cells treated for 15 min with 50 μM NGF(1-14) peptides or 50 ng/mL NGF protein in the absence (☐) or in the presence of 1 μM CuSO_4_ (■) or in 50 μM BCS 24 h pre-treated medium (■). Data obtained from at least three independent experiments. The phosphorylated level of Erk1/2 and Akt was reported as ratio over the corresponding total protein (*** *p* < 0.001 versus control; §§§ *p* < 0.001 versus CuSO_4_; ### *p* < 0.001 versus BCS; one-way Anova).

Figure 7 shows the effect of d-NGF(1-15) (50 μM) on Erk1/2 and Akt phosphorylation after 15 min of treatment; d-NGF(1-14) increased the level of pErk1/2 (383 ± 24%) significantly more than NGF(1-14) (311 ± 14%), while phosphorylation of Ser-473 Akt did not show any significant difference between NGF(1-14) (180 ± 14%) and d-NGF(1-15) (207 ± 5%). The treatment in the presence of 1 μM CuSO_4_ showed a similar trend, with phosphorylation of Erk1/2 significantly higher with d-NGF(1-15) (447 ± 11%) than with NGF(1-14) (370 ± 20%), while the level of pAkt did not show any significant difference between NGF(1-14) (228 ± 13%) and d-NGF(1-15) (239 ± 9%).

The deprivation of copper ions determined by BCS (50 μM) addition decreased the phosphorylation of Akt due to d-NGF(1-15) (150 ± 17%) and NGF(1-14) (119 ± 25%). The effect was more evident for Erk1/2 (d-NGF(1-15) (260 ± 31%); NGF(1-14) (207 ± 22%).

NGF alone increased the phosphorylation of Akt (255 ± 12%) and Erk1/2 (545 ± 27%), as it also did in the presence of copper (Akt: 341% ± 13 and Erk1/2: 581% ± 31). The treatment with NGF in the presence of BCS decreased the effect of protein (Akt: 172 ± 48% and Erk1/2: 311 ± 16%). 

**Figure 8:**
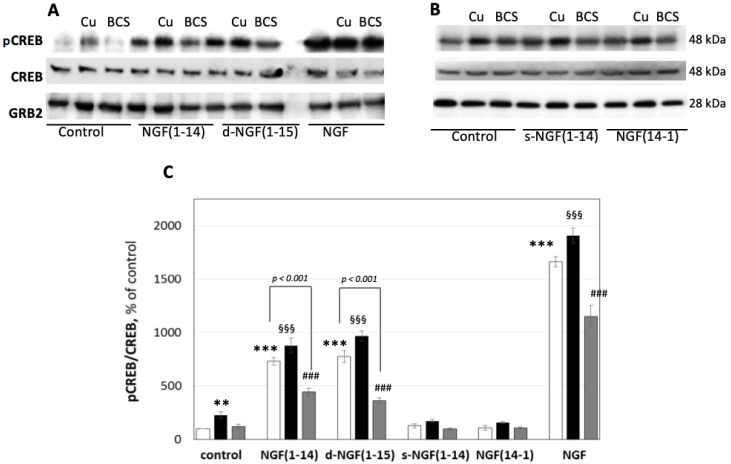
Effect of NGF or NGF peptides on AMP response element-binding protein (CREB) phosphorylation. Representative western blotting (**A**,**B**) and densitometric analyses (**C**) of pCREB in PC12 cells treated for 15 min with 50 μM NGF peptides or 50 ng/mL NGF protein in the absence (☐) or in the presence of 1 μM CuSO_4_ (■) or in 50 μM BCS 24 h pre-treated medium (■). Data obtained from at least three independent experiments. The phosphorylated level of CREB was reported as ratio over the corresponding total protein (** *p* < 0.01, *** *p* < 0.001 versus control; §§§ *p* < 0.001 versus CuSO_4_; ### *p* < 0.001 versus BCS; one-way Anova).

Copper (II) ion significantly influenced not only the tyrosine kinase-mediated pathways but also the phosphorylation of CREB, as shown in the following. It is important to note that the presence of just 1 μM of CuSO_4_ increased the level of pCREB (up to 229 ± 29%) (Figure 8). The d-NGF(1-15) was able to activate CREB through Ser133 phosphorylation, as previously reported for NGF(1-14).^18^ The level of pCREB after 30 min of incubation with NGF(1-14) (731 ± 36%) and d-NGF(1-15) (776 ± 55%) significantly increased after the addition of CuSO_4_ (1 µM), both for NGF(1-14) (880 ± 68%) and d-NGF(1-15) (970 ± 49%), respectively. The presence of BCS during the treatment decreased the effect of NGF(1-14) (443 ± 36%) and d-NGF(1-15) (363 ± 25%), respectively. The inhibitory effect of BCS on CREB phosphorylation at Ser133 stressed the synergic effect of the metal ion. The cell treatment by s-NGF(1-14) (129 ± 19%) or the NGF(14-1) (110 ± 23%) did not affect the CREB phosphorylation. The addition of CuSO_4_ induced a significant increase of pCREB for both s-NGF(1-14) (172 ± 14%) and NGF(14-1) (158 ± 11%), respectively, but was still lower than that caused by metal ion alone.

### 3.8. Copper(II) Ion, NGF(1-14) and d-NGF(1-15) Induce BDNF Secretion

pCREB modulates the transcription of genes involved in synaptic plasticity and memory formation [72], such as BDNF [73]. BDNF is essential for growth, survival, and neuronal cell differentiation, and has been invoked in the pathophysiology of Alzheimer’s disease, Huntington’s disease, and Parkinson’s disease [74].

**Figure 9:**
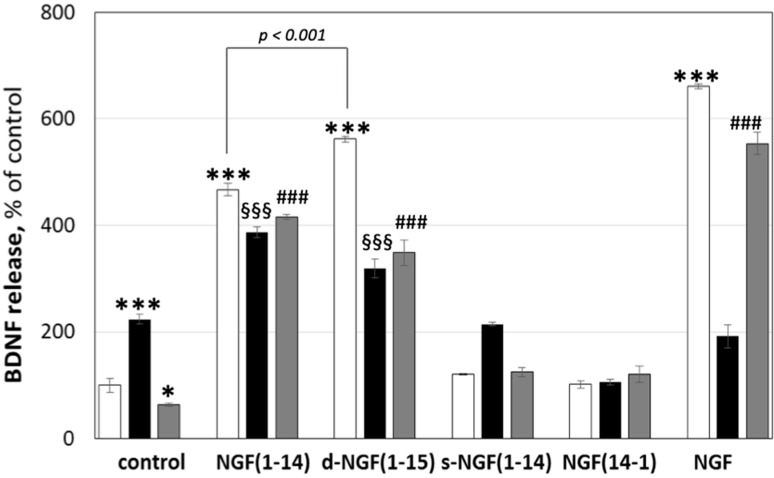
Effect of NGF and NGF(1-14) peptides on the brain-derived neurotrophic factor (BDNF) secretion. Starved PC12 cells were stimulated for 24 h with 50 ng/mL of NGF or 10 μM of NGF(1-14) peptides in the absence (☐) or in the presence of CuSO_4_ (■) or BCS (■). Cell culture mediums were collected and analyzed by ELISA BDNF kit. Data obtained from at least three independent experiments (* *p* < 0.05, *** *p* < 0.001 versus control; §§§ *p* < 0.001 versus CuSO_4_; ### *p* < 0.001 versus BCS; one-way Anova).

Figure 9 shows the increase of BDNF levels after the treatment with CuSO_4_ up to 224 ± 9%. NGF(1-14) and d-NGF(1-15) increased BDNF levels compared to the untreated control up to 467 ± 11% and 561 ± 5%, respectively.

NGF further amplified the release of BDNF up to 660 ± 4%. CuSO_4_ addition slightly decreased the effect of the NGF mimicking peptides and, to a greater extent, that of NGF. s-NGF(1-14) or NGF(14-1) did not affect BDNF release, while metal addition influenced only the behavior of s-NGF(1-14), which showed the same effect of Cu^2+^ alone.

### 3.9. NGF(1-14) and d-NGF(1-15) Inhibits Protein Tyrosine-Phosphatase Activity

Protein tyrosine phosphatases are key regulators of cellular phosphorylation signaling [75]. Recently, copper and its mono and dinuclear complexes have been shown to inhibit the activity of these enzymes [76]. Previously, we attributed the inhibition effect of the NGF(1-14) in the presence of copper to the intracellular copper(I) interaction with the active site cysteine thiolate of tyrosine phosphatase [21] with consequent stimulus of tyrosine phosphorylation as it was herein found for d-NGF(1-15). In keeping with previous findings, we determined the tyrosine phosphorylation of the whole protein lysate in PC12 cells stimulated with the NGF(1-14) peptides in the presence or in the absence of 1 µM CuSO_4_ or 50 µM BCS.

**Figure 10:**
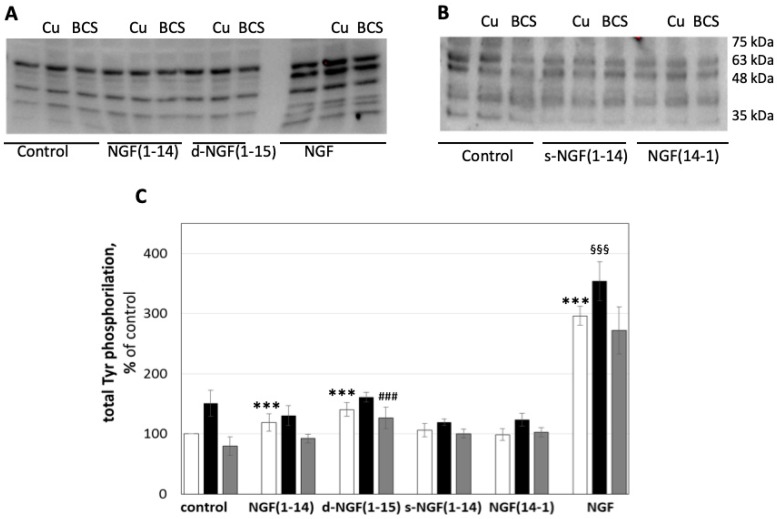
Effect of NGF and NGF(1-14) peptides on protein tyrosine phosphatase activity. Representative western blotting (**A**,**B**) and densitometric analyses (**C**) of total tyrosine phosphorylation in PC12 cells treated for 15 min with 50 μM NGF(1-14) peptides or NGF protein in the absence (☐) or in the presence of 1 μM CuSO_4_ (■) or 50 μM BCS 24 h pre-treated medium (■). Data obtained from at least three independent experiments. (*** *p* < 0.001 versus control; §§§ *p* < 0.001 versus CuSO_4_; ### *p* < 0.001 versus BCS; one-way Anova).

The results in Figure 10 show that tyrosine phosphorylation increased upon treatment with NGF(1–14) (125 ± 14%), d-NGF(1–15) (136 ± 11%), and NGF (296 ± 16%) in comparison to the unstimulated control. As expected, Cu^2+^ addition increased the total tyrosine phosphorylation, both alone (145 ± 22%) and in synergy with the ligands [134 ± 16% for NGF(1-14) and 157 ± 8% for d-NGF(1-15), respectively]. On the other hand, the chelating agent BCS counteracted this effect and decreased the phosphorylation process (82 ± 15%), also in the presence of NGF(1-14) (91 ± 7%) or d-NGF(1-15) (120 ± 18%). Both s-NGF(1-14) (106 ± 11%) and NGF(14-1) (99 ± 10%) were unable to induce a statistically significant increase of total tyrosine phosphorylation. However, the cells incubated with the addition of 1 µM copper increased the tyrosine phosphorylation for both s-NGF(1-14) (119 ± 5%) and NGF(14-1) (124 ± 11%), respectively.

The results clearly support the previous hypothesis on the effect of metal ion to stimulate tyrosine phosphorylation. The peptides and NGF contribute at the process in a way that appears to be directly related to their ability to bind copper.

This finding also suggests a dual role of metal ions—on the one hand, it supports the molecular recognition of NGF mimetic peptides and NGF with the receptor, and on the other hand, it utilizes the ionophore ability of the peptides to act at the intracellular level.

### 3.10. NGF(1-14) and d-NGF(1-15) Peptides Display Different Trafficking and Ionophore Activity in PC12 Cells

**Figure 11:**
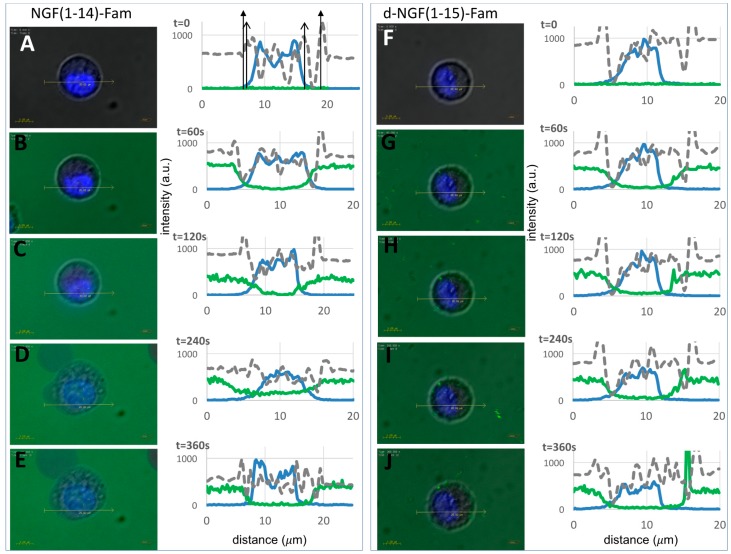
Live cell imaging by confocal microscopy of Fam-labeled NGF(1-14) and d-NGF(1-15) internalization. Merged optical bright field (grey) and confocal (blue: nuclear staining, λ_ex/em_ = 405/425–475 nm; green: Fam, λ_ex/em_ = 488/500–530 nm) micrographs and corresponding section analysis for PC12 before (**A**,**F**) and after (60 s, 120 s, 240 s, 360 s) the addition of 10 μM NGF(1-14)-Fam (**B**–**E**) or 10 μM d-NGF(1-15)-Fam (**G**–**J**), respectively. The solid and open arrows in the top left plot guide the eye for the cell and nuclear membranes, respectively.

We used real-time live cell imaging by confocal microscopy to test the intracellular trafficking of d-NGF(1-15) in comparison with NGF(1-14), which was previously demonstrated to have ionophore activity [21]. Figure 11 demonstrates a quick internalization of the Fam-labeled peptides by PC12 live cells. The merged confocal fluorescence (i.e., the green emission from Fam and the blue fluorescence of Hoechst-stained nuclei) and bright field (in grey) micrographs permitted to localize the peptide, both extracellularly and intracellularly, to track the cellular uptake/efflux processes. Specifically, the overlapped intensity profile curves for the three channels (green, blue, and grey) measured along a 20 μm-long traced line across the cell allowed visualization of the spatial shifts of the peptides with reference to the cell membrane, cytoplasm, and nucleus, respectively. The time sequence displays a representative cell imaged immediately before the addition of NGF(1-14) (Figure 11A) or d-NGF(1-15) (Figure 11F), and four subsequent micrographs for each peptide recorded in the time range of 1-6 min after the addition of a 10 μM concentration of monomer (Figure 11, panels B–E) or dimer (Figure 11, panels G-J) peptides. 

NGF(1-14)-Fam quickly entered in the cytoplasm (Figure 11B,C), accumulated into the nucleus (Figure 11D), and effluxed from the cell (Figure 11E). As for d-NGF(1-15)-Fam, it showed an uptake profile similar to that of NGF(1-14)-Fam, with the green fluorescence detected in the cytoplasm and in proximity to the nuclear membrane (Figure 11G). Differently than the monomer, the dimer molecules showed the tendency to form aggregates (Figure 11H,I), which preferentially gathered at the cell membrane as the peptide effluxed from the cell (Figure 11J).

**Figure 12:**
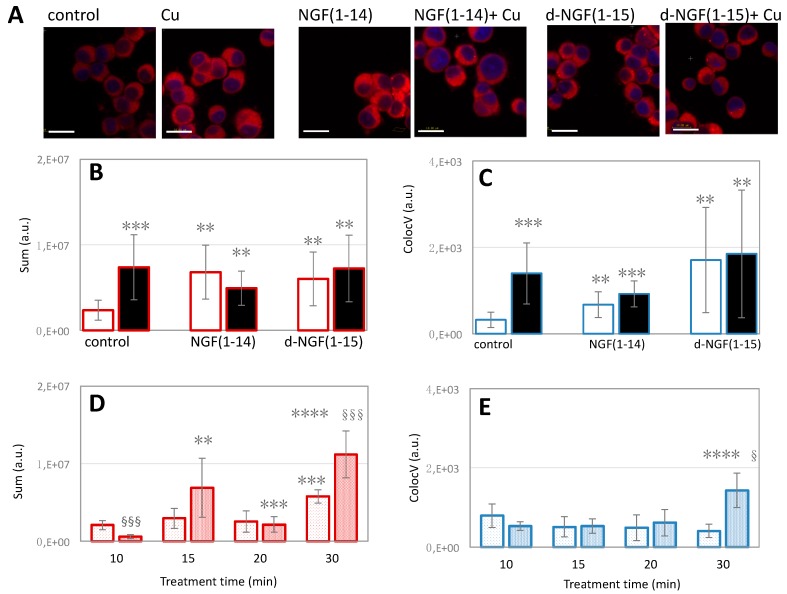
The effect of d-NGF(1-15) and NGF(1-14) peptides on copper homeostasis. In (**A**): representative merged confocal microscopy images of coppersensor-1 (red) and Hoechst33342 ( blue) stained cells after 15 min of treatment with 10 μM NGF(1-14) or d-NGF(1-15), either in the absence or in the presence of 1 µM CuSO_4_. Untreated and 1 µM CuSO_4_ treated-cells are included as negative and positive controls, respectively (scale bar = 10 μm). (**B**,**C)**: Quantitative analysis of the confocal micrographs for total cytoplasmic Cu^+^ (**B**) and nuclear Cu^+^ (**C**) in the cells incubated with the peptides in basal medium (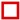, 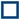) or copper-supplemented medium (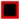, 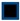). In (**D**,**E**): quantitative analyses of total intracellular copper (**D**) or nuclear-confined copper (**E**) after PC12 cells incubation for 10, 15, 20 or 30 min with 10 μM NGF(1-14) (cytoplasm: 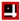; nuclei: 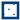) or 10 μM d-NGF(1-14) (cytoplasm: 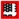; nuclei: 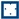). ** *p* < 0.01, *** *p* < 0.001, versus control.; ** *p* < 0.01, *** *p* < 0.001, **** *p* < 0.0001 versus the preceding incubation time; § *p* < 0.05, §§§ *p* < 0.001 versus [NGF(1-14)].

To correlate the peptide influx/efflux with the intracellular copper levels, cells were stained with the copper sensor-1 probe, a selective and specific sensor of monovalent Cu [77]. Figure 12 shows that d-NGF(1-15) had an ionophore capability similar to that of NGF(1-14) [21], as evidenced by quantitative analyses of total cytoplasmic and nuclear Cu^+^ (corresponding to the red fluorescence of the copper sensor-1 probe) for the cells incubated with the peptides, both in the basal medium and in the medium supplemented with CuSO_4_. 

A statistically significant increase of intracellular copper was observed in the cells treated with the peptides (10 μM) for 15 min with respect to the control cells in basal medium (Figure 12A–C). This finding suggested that the peptide extracellularly bound the divalent copper typically present in the basal medium at concentrations up to 1 μM and could therefore act as an ionophore during its uptake/efflux from the cell. It must be noted that such an increase of intracellular copper levels, measured both in the cytoplasm (Figure 12B) and in the nuclei (Figure 12C), was not statistically different than that found in the cells incubated with CuSO_4_ (1 μM)-supplemented medium as well as that of the cells incubated with the pre-formed copper-peptides complexes (10:1 of peptide to metal mole ratio).

Another interesting finding was obtained from the quantitative analysis of intracellular Cu^+^ levels, both in the total cytoplasm and in the nuclei, for PC12 cells treated 10 min, 15 min, 20 min, or 30 min with 10 μM NGF(1-14) or 10 μM d-NGF(1-14) (Figure 12D,E). Different dynamics of peptide-perturbed copper trafficking occurred for d-NGF(1-15) and NGF(1-14). In fact, two relative maxima in the cytoplasmic Cu^+^ levels were found respectively at 15 min and 30 min after the treatment with d-NGF(1-15) (Figure 12D), whereas the treatment with NGF(1-14) displayed a maximum Cu^+^ level in the cytoplasm only after 30 min of cells incubation with the peptide (Figure 12E). 

### 3.11. NGF1-14 Retains the Ability of the Full-Length Wild-Type NGF in Suppressing the Degeneration of Primary NGF-Target Neurons

In order to ascertain that NGF(1-14) peptides were also biologically active in post-mitotic cellular environment, by inducing physiologically significant responses that resemble those evoked by its whole parental counterpart in terminally-differentiated primary neurons, we took advantage of a well-characterized NGF-dependent in vitro neuronal model such as cholinergic septo-hippocampal cultures [51]. 

In this system, NGF withdrawal induced a reversible, time-dependent, “dying-back” process of TrkA-dependent presynaptic degeneration, leading to an early and progressive loss in selected presynaptic and vesicles trafficking proteins (including synapsin I, SNAP-25, α-synuclein), which in turn negatively impacted the excitatory neurotransmission [78]. The ability of NGF(1-14) in blocking the “dying-back” mechanism(s) of mature cholinergic primary neurons by compensating the loss of presynaptic markers caused by 6 h of NGF deprivation was assessed as preliminary results of the behavior of the NGF peptides in different neuronal cell line.

To this aim, septal cholinergic-enriched cultures grown continuously in 0.2% B27 media in the presence of exogenous NGF (100 ng/mL) from plating were deprived of their trophic support for six hours. At this time point, cell were either harvested (6h) or re-exposed to NGF (100 ng/mL) and NGF(1-14) and further kept up to 24 h, when they were eventually collected (6h+NGF; 6h+NGF(1-14)). By Western blotting analysis on whole-cell lysates (Figure S3), we found out that the loss in protein amounts of synapsin I following 6h NGF starvation was significantly suppressed by the external application of increasing concentration of NGF(1-14) (5–10 μM) in a dose-dependent manner (** *p* < 0.01, *** *p* < 0.0001 versus −6h, respectively), mimicking the rescue action of the wild-type NGF (* *p* < 0.05 versus −6h). Similar results were also found for SNAP-25 (* *p* < 0.05, **p < 0.01 versus −6h, respectively) and α-synuclein (data not shown), the other two presynaptic proteins whose expression levels we previously reported to be strongly suppressed in −6h NGF-deprived cultures by *de novo* external re-application of NGF [51]. Taken togethr, these findings demonstrate that NGF(1-14) is endowed with the same functional phenotype of its wild-type holoprotein and is able, in vitro, to control the cholinergic presynaptic stability of mature primary neurons, just as reported for full-length NGF [51].

## 4. Conclusions

Experimental and in silico approaches allowed us to disclose NGF mimetic peptides encompassing the N-terminal domain of the whole protein NGF. In particular, we found that the newly synthesized peptide d-NGF(1-15) encompassing two NGF N-terminal monomeric units NGF(1-14) interacted with the TrkA-D5 domain and induced the activation of its signaling pathways. Differently, both the peptides encompassing the reverse NGF(14-1) and the scrambled s-NGF(1-14) amino acid sequences did not induce differentiation nor proliferation in PC12 cells, confirming the dependence of biological activity on the specific primary sequence.

Spectroscopic findings indicate that the copper(II) binding to NGF(1-14) and d-NGF(1-15) stabilized the secondary structure of the peptides, suggesting a strengthening of the noncovalent interactions that allowed for the molecular recognition of D5 domain of TrkA and the activation of the signaling pathways. At the same time, the metal ion inhibited the tyrosine phosphatase activity and potentiated the kinase cascades by means of the ionophoric features of the NGF mimetic peptides. 

The greater molecular recognition of TrkA-D5 by the dimeric peptide d-NGF(1-15) enhanced the receptor phosphorylation at Tyr490 more than NGF(1-14), activating the MAPK–ERK and PI3K–AKT pathways, whereas the phosphorylation of Tyr785 was not induced, and the associated activation of the PLCγ–PKC signaling pathway was not found (data not shown). 

It was remarkable that, contrary to the NGF protein, the interaction of NGF(1-14) and d-NGF(1-15) peptides with metal ion favored TrkA receptor internalization, whereas these peptides, both in the absence and in the presence of copper ions, did not bind p75 and did not induce p75 internalization. The use of neurotrophins as therapeutic tools has encountered significant contraindications because of pleiotropic mechanisms of actions due to p75 activation, and NGF(1-14) and d-NGF(1-15) might resolve this problem. 

Both in vitro cell cultures and in vivo studies indicate that ionophore ligands or chelating molecules with the capacity to increase intracellular metal bioavailability can activate neuroprotective cell signaling pathways pertinent to Alzheimer’s disease, resulting in upregulation of matrix metalloprotease (MMP) activity and degradation of amyloid-β [79,80].

Up-regulation of MMP by Cu^2+^ or Zn^2+^ has been previously reported [81], although excess Zn^2+^ can also inhibit MMP activity [82]. Hwang et al. reported that Cu^2+^ increases the activity of matrix metalloproteinase (MMP2 and MMP9) in cortical neurons, resulting in the release of pro-BDNF and BDNF and activation of TrkB [83], analogous to the behavior previously found for Zn^2+^. Furthermore, the release of the two metal ions at the synapses during neuronal activity may contribute to synaptic plasticity [84,85].

d-NGF(1-15) and NGF(1-14) were able to activate the BDNF release and increase the effect of Cu^2+^ alone, suggesting that their ionophore ability favors an intracellular effect of the metal ion. Cu^2+^ alone could activate the BDNF maturation in a MMP-dependent mode at extracellular levels, as previous mentioned [83]. Notably, MMP levels could be increased through the stimulation of the PI3K-Akt and MAPK (ERK) pathways, which was consistent with our findings in NGF(1-14) and d-NGF(1-15)-Cu^2+^ treated cells [86,87]. Furthermore, the Cu^2+^ ion appeared to decrease the ability of the two peptides in a way directly related to their affinity with the metal ion, affecting d-NGF(1-15) more than NGF(1-14). NGF appeared to reduce its activity binding Cu^2+^ with a larger affinity than the two peptides; this strong decrease in BDNF release further suggested that the metal ion also present in the medium contributed to the neurotrophin release at extracellular level.

Considering that MMP causes the production of mature neurotrophins, we can speculate that Cu^2+^ and its complex with NGF mimetic peptides contribute to counteract the differential deregulation of NGF and BDNF found in Alzheimer’s disease [88].

Finally, the previously hypothesized copper ability to inhibit tyrosine phosphatase activity [21] was validated, suggesting that, in the presence of the metal ion, the NGF mimics not only imitate the signaling effect of NGF but also add further favorable pathways at the neurotrophin mode of action.

Intriguingly, the signaling cascade induced by the NGF peptides ultimately involved CREB activation and increase in BDNF protein level, keeping with our previous result showing an increase of BDNF mRNA [21]. Moreover, NGF(1-14) imitated the full-length NGF by blocking the “dying-back” presynaptic degeneration in cholinergic septo-hippocampal neurons suppressing the loss of synapsinI that was involved in the presynaptic actions of BDNF at central nerve terminals [78]. 

Neurotrophins and their receptors have overlapping expression patterns and functions in many areas of the brain, but some neural mechanisms are modulated by specific systems, including NGF and BDNF. In animal models of the disease and in humans, the levels of NGF and BDNF follow different trajectories in brain areas affected in Alzheimer’s disease [88,89]. 

While transgenic rats exhibit no change in NGF mRNA, even in the presence of amyloid protein deposition, BDNF mRNA expression is reduced starting at very early stages when no plaques are present [90]. The same trend has been found in subjects with Alzheimer’s disease where BDNF mRNA, its precursor (proBDNF), and mature protein levels significantly decrease, while NGF mRNA does not change, and the NGF precursor molecule proNGF increases [91,92].

We can speculate that the NGF peptides can act as dual agents against the disease, not only restoring NGF, TrkA-dependent, and p75-independent activities, but also increasing BDNF levels [93].

All these promising connections can pave the way for a bespoke development of interesting novel drugs for neurodegenerative diseases.

## Figures and Tables

**Table 1:** UV-Vis and circular dichroism (CD) spectroscopic parameters of copper(II) complexes with peptides at pH = 7.4. Metal to ligand molar ratio 1:1, [L] = 1 × 10^−3^ M.

Peptide	λ/nm (ε/M^−1^ cm^−1^)	λ/nm (Δε/M^−1^ cm^−1^)
NGF(1-14) ^a^	605 (104)	284 (−1.40), 328 (0.55), 340 (0.22), 632 (−0.58)
d-NGF(1-15)	590 (130)	288 (−3.18), 322 (1.15), 500 (0.11), 587 (−0.58)
s-NGF(1-14)	618 (85)	288 (−1.150), 324 (0.70), 599 (−0.57)
NGF(14-1)	615 (120)	282 (−0.42), 316 (−0.32), 354 (0.07); 515 (0.31), 626 (−0,62)

^a^ reference [48].

## Supplementary Materials

The following are available online at https://www.mdpi.com/2073-4409/8/4/301/s1, Figure S1: UV-vis CD spectra of copper complexes of NGF peptides, Figure S2: Far-UV CD spectra of copper complexes of NGF peptides, Figure S3: western blot and densitometric quantification of synapsin I and synaptosomal-associated protein 25 on septal cholinergic-enriched cultures treated with NGF and NGF(1-14).

## References

[1] Skaper S.D. (2012). The Neurotrophin Family of Neurotrophic Factors: An Overview. Neurotrophic Factors.

[2] Bothwell M. (2014). NGF, BDNF, NT3, and NT4. Neurotrophic Factors.

[3] Barbacid M. (1995). Neurotrophic factors and their receptors. Curr. Opin. Cell Biol..

[4] Ullrich A., Schlessinger J. (1990). Signal transduction by receptors with tyrosine kinase activity. Cell.

[5] Underwood C.K., Coulson E.J. (2008). The p75 neurotrophin receptor. Int. J. Biochem. Cell Biol..

[6] Cohen S., Levi-Montalcini R., Hamburger V. (1954). A Nerve Growth-Stimulating Factor Isolated from Sarcom as 37 and 180. Proc. Natl. Acad. Sci. USA.

[7] Berg M.M., Sternberg D.W., Hempstead B.L., Chao M.V. (1991). The low-affinity p75 nerve growth factor (NGF) receptor mediates NGF-induced tyrosine phosphorylation. Proc. Natl. Acad. Sci. USA.

[8] Chao M.V. (2003). Neurotrophins and their receptors: A convergence point for many signaling pathways. Nat. Rev. Neurosci..

[9] Schneider R., Schweiger M. (1991). A novel modular mosaic of cell adhesion motifs in the extracellular domains of the neurogenic trk and trkB tyrosine kinase receptors. Oncogene.

[10] Blum R., Konnerth A. (2005). Neurotrophin-Mediated Rapid Signaling in the Central Nervous System: Mechanisms and Functions. Physiology.

[11] Zaccaro M.C., Ivanisevic L., Perez P., Meakin S.O., Saragovi H.U. (2001). p75 Co-receptors Regulate Ligand-dependent and Ligand-independent Trk Receptor Activation, in Part by Altering Trk Docking Subdomains. J. Biol. Chem..

[12] Skaper S.D. (2008). The biology of neurotrophins, signaling pathways, and functional peptide mimetics of neurotrophins and their receptors. CNS Neurol. Disord. Drug Targets.

[13] Peleshok J., Saragovi H.U. (2006). Functional mimetics of neurotrophins and their receptors. Biochem. Soc. Trans..

[14] Saragovi H.U., Gehring K. (2000). Development of pharmacological agents for targeting neurotrophins and their receptors. Trends Pharmacol. Sci..

[15] Longo F.M., Massa S.M. (2013). Small-molecule modulation of neurotrophin receptors: A strategy for the treatment of neurological disease. Nat. Rev. Drug Discov..

[16] Kazim S.F., Iqbal K. (2016). Neurotrophic factor small-molecule mimetics mediated neuroregeneration and synaptic repair: Emerging therapeutic modality for Alzheimer’s disease. Mol. Neurodegener..

[17] Josephy-Hernandez S., Jmaeff S., Pirvulescu I., Aboulkassim T., Saragovi H.U. (2017). Neurotrophin receptor agonists and antagonists as therapeutic agents: An evolving paradigm. Neurobiol. Dis..

[18] Coulson E.J., Simmons D.A., Knowles J.K., Belichenko N.P., Banerjee G., Finkle C., Massa S.M., Longo F.M. (2014). A Small Molecule p75NTR Ligand, LM11A-31, Reverses Cholinergic Neurite Dystrophy in Alzheimer’s Disease Mouse Models with Mid- to Late-Stage Disease Progression. PLoS ONE.

[19] Habtemariam S. (2018). The brain-derived neurotrophic factor in neuronal plasticity and neuroregeneration: New pharmacological concepts for old and new drugs. Neural Regen. Res..

[20] Travaglia A., Pietropaolo A., Di Martino R., Nicoletti V.G., La Mendola D., Calissano P., Rizzarelli E. (2015). A Small Linear Peptide Encompassing the NGF N-Terminus Partly Mimics the Biological Activities of the Entire Neurotrophin in PC12 Cells. ACS Chem. Neurosci..

[21] Pandini G., Satriano C., Pietropaolo A., Gianì F., Travaglia A., La Mendola D., Nicoletti V.G., Rizzarelli E. (2016). The Inorganic Side of NGF: Copper(II) and Zinc(II) Affect the NGF Mimicking Signaling of the N-Terminus Peptides Encompassing the Recognition Domain of TrkA Receptor. Front. Neurosci..

[22] Finkbeiner S., Tavazoie S.F., Maloratsky A., Jacobs K.M., Harris K.M., Greenberg M.E. (1997). CREB: A major mediator of neuronal neurotrophin responses. Neuron.

[23] Travaglia A., Pietropaolo A., La Mendola D., Nicoletti V.G., Rizzarelli E. (2012). The inorganic perspectives of neurotrophins and Alzheimer’s disease. J. Inorg. Biochem..

[24] Kheirvari S., Uezu K., Yamamoto S., Nakaya Y. (2008). High-dose dietary supplementation of vitamin A induces brain-derived neurotrophic factor and nerve growth factor production in mice with simultaneous deficiency of vitamin A and zinc. Nutr. Neurosci..

[25] Allington C., Shamovsky I.L., Ross G.M., Riopelle R.J. (2001). Zinc inhibits p75NTR-mediated apoptosis in chick neural retina. Cell Death Differ..

[26] Birkaya B., Aletta J.M. (2005). NGF promotes copper accumulation required for optimum neurite outgrowth and protein methylation. J. Neurobiol..

[27] Bica L., Liddell J.R., Donnelly P.S., Duncan C., Caragounis A., Volitakis I., Paterson B.M., Cappai R., Grubman A., Camakaris J. (2014). Neuroprotective copper bis(thiosemicarbazonato) complexes promote neurite elongation. PLoS ONE.

[28] Ross G.M., Shamovsky I.L., Lawrance G., Solc M., Dostaler S.M., Jimmo S.L., Weaver D.F., Riopelle R.J. (1997). Zinc alters conformation and inhibits biological activities of nerve growth factor and related neurotrophins. Nat. Med..

[29] Maitra R., Shamovsky I.L., Wang W., Solc M., Lawrance G., Dostaler S.M., Ross G.M., Riopelle R.J. (2000). Differential effects of transition metal cations on the conformation and biological activities of nerve growth factor. Neurotox. Res..

[30] Wang J.K. (1999). Cu2+ induces Ca2+-dependent neurotransmitter release from brain catecholaminergic nerve terminals. Eur. J. Pharmacol..

[31] Maliartchouk S., Debeir T., Beglova N., Cuello A.C., Gehring K., Saragovi H.U. (2000). Genuine monovalent ligands of TrkA nerve growth factor receptors reveal a novel pharmacological mechanism of action. J. Biol. Chem..

[32] Colangelo A.M., Bianco M.R., Vitagliano L., Cavaliere C., Cirillo G., De Gioia L., Diana D., Colombo D., Redaelli C., Zaccaro L. (2008). A new nerve growth factor-mimetic peptide active on neuropathic pain in rats. J. Neurosci..

[33] Marte A., Messa M., Benfenati F., Onofri F. (2016). Synapsins Are Downstream Players of the BDNF-Mediated Axonal Growth. Mol. Neurobiol..

[34] Peng S., Li W., Lv L., Zhang Z., Zhan X. (2018). BDNF as a biomarker in diagnosis and evaluation of treatment for schizophrenia and depression. Discov. Med..

[35] Yang Y., Liu Y., Wang G., Hei G., Wang X., Li R., Li L., Wu R., Zhao J. (2019). Brain-derived neurotrophic factor is associated with cognitive impairments in first-episode and chronic schizophrenia. Psychiatry Res..

[36] Notaras M., Hill R., Gogos J.A., van den Buuse M. (2015). BDNF Val66Met genotype determines hippocampus-dependent behavior via sensitivity to glucocorticoid signaling. Mol. Psychiatry.

[37] Beck M., Flachenecker P., Magnus T., Giess R., Reiners K., Toyka K.V., Naumann M. (2009). Autonomic dysfunction in ALS: A preliminary study on the effects of intrathecal BDNF. Amyotroph. Lateral Scler..

[38] Essmann U., Perera L., Berkowitz M.L., Darden T., Lee H., Pedersen L.G. (1995). A smooth particle mesh Ewald method. J. Chem. Phys..

[39] Hornak V., Abel R., Okur A., Strockbine B., Roitberg A., Simmerling C. (2006). Comparison of multiple Amber force fields and development of improved protein backbone parameters. Proteins: Struct. Funct. Bioinform..

[40] Jorgensen W.L., Chandrasekhar J., Madura J.D., Impey R.W., Klein M.L. (1983). Comparison of simple potential functions for simulating liquid water. J. Chem. Phys..

[41] Miyamoto S., Kollman P.A. (1992). Settle: An analytical version of the SHAKE and RATTLE algorithm for rigid water models. J. Comput. Chem..

[42] Hess B., Kutzner C., van der Spoel D., Lindahl E. (2008). GROMACS 4: Algorithms for Highly Efficient, Load-Balanced, and Scalable Molecular Simulation. J. Chem. Theory Comput..

[43] Bussi G., Donadio D., Parrinello M. (2007). Canonical sampling through velocity rescaling. J. Chem. Phys..

[44] Rosta E., Buchete N.V., Hummer G. (2009). Thermostat artifacts in replica exchange molecular dynamics simulations. J. Chem. Theory Comput..

[45] Daura X., Gademann K., Jaun B., Seebach D., van Gunsteren W.F., Mark A.E. (1999). Peptide Folding: When Simulation Meets Experiment. Angew. Chem. Int. Ed..

[46] Wiesmann C., Ultsch M.H., Bass S.H., de Vos A.M. (1999). Crystal structure of nerve growth factor in complex with the ligand-binding domain of the TrkA receptor. Nature.

[47] De Vries S.J., van Dijk M., Bonvin A.M. (2010). The HADDOCK web server for data-driven biomolecular docking. Nat. Protoc..

[48] Travaglia A., Arena G., Fattorusso R., Isernia C., La Mendola D., Malgieri G., Nicoletti V.G., Rizzarelli E. (2011). The Inorganic Perspective of Nerve Growth Factor: Interactions of Cu2+ and Zn2+ with the N-Terminus Fragment of Nerve Growth Factor Encompassing the Recognition Domain of the TrkA Receptor. Chem. - A Eur. J..

[49] Forte G., Travaglia A., Magri A., Satriano C., La Mendola D. (2014). Adsorption of NGF and BDNF derived peptides on gold surfaces. Phys. Chem. Chem. Phys..

[50] La Mendola D., Farkas D., Bellia F., Magri A., Travaglia A., Hansson O., Rizzarelli E. (2012). Probing the copper(II) binding features of angiogenin. Similarities and differences between a N-terminus peptide fragment and the recombinant human protein. Inorg. Chem..

[51] Latina V., Caioli S., Zona C., Ciotti M.T., Amadoro G., Calissano P. (2017). Impaired NGF/TrkA Signaling Causes Early AD-Linked Presynaptic Dysfunction in Cholinergic Primary Neurons. Front. Cell. Neurosci..

[52] Troncone G., Walter R.F.H., Werner R., Vollbrecht C., Hager T., Flom E., Christoph D.C., Schmeller J., Schmid K.W., Wohlschlaeger J. (2016). ACTB, CDKN1B, GAPDH, GRB2, RHOA and SDCBP Were Identified as Reference Genes in Neuroendocrine Lung Cancer via the nCounter Technology. Plos ONE.

[53] Miller E.W., Zeng L., Domaille D.W., Chang C.J. (2006). Preparation and use of Coppersensor-1, a synthetic fluorophore for live-cell copper imaging. Nat. Protoc..

[54] Settanni G., Cattaneo A., Carloni P. (2003). Molecular Dynamics Simulations of the NGF-TrkA Domain 5 Complex and Comparison with Biological Data. Biophys. J..

[55] Wehrman T., He X., Raab B., Dukipatti A., Blau H., Garcia K.C. (2007). Structural and Mechanistic Insights into Nerve Growth Factor Interactions with the TrkA and p75 Receptors. Neuron.

[56] Lessmann V., Gottmann K., Malcangio M. (2003). Neurotrophin secretion: Current facts and future prospects. Prog. Neurobiol..

[57] Scarpi D., Cirelli D., Matrone C., Castronovo G., Rosini P., Occhiato E.G., Romano F., Bartali L., Clemente A.M., Bottegoni G. (2012). Low molecular weight, non-peptidic agonists of TrkA receptor with NGF-mimetic activity. Cell Death Dis..

[58] Satriano C., Forte G., Magri A., Di Pietro P., Travaglia A., Pandini G., Giani F., La Mendola D. (2016). Neurotrophin-mimicking peptides at the biointerface with gold respond to copper ion stimuli. Phys. Chem. Chem. Phys..

[59] Sóvágó I., Ősz K. (2006). Metal ion selectivity of oligopeptides. Dalton Trans..

[60] La Mendola D., Arnesano F., Hansson O., Giacomelli C., Calo V., Mangini V., Magri A., Bellia F., Trincavelli M.L., Martini C. (2016). Copper binding to naturally occurring, lactam form of angiogenin differs from that to recombinant protein, affecting their activity. Metallomics.

[61] La Mendola D., Magri A., Campagna T., Campitiello M.A., Raiola L., Isernia C., Hansson O., Bonomo R.P., Rizzarelli E. (2010). A doppel alpha-helix peptide fragment mimics the copper(II) interactions with the whole protein. Chemistry.

[62] La Mendola D., Magri A., Santoro A.M., Nicoletti V.G., Rizzarelli E. (2012). Copper(II) interaction with peptide fragments of histidine-proline-rich glycoprotein: Speciation, stability and binding details. J. Inorg Biochem.

[63] Bellia F., La Mendola D., Maccarrone G., Mineo P., Vitalini D., Scamporrino E., Sortino S., Vecchio G., Rizzarelli E. (2007). Copper(II) complexes with β-cyclodextrin–homocarnosine conjugates and their antioxidant activity. Inorg. Chim. Acta.

[64] Ross G.M., Shamovsky I.L., Woo S.B., Post J.I., Vrkljan P.N., Lawrance G., Solc M., Dostaler S.M., Neet K.E., Riopelle R.J. (2001). The binding of zinc and copper ions to nerve growth factor is differentially affected by pH: Implications for cerebral acidosis. J. Neurochem..

[65] White A.R., Barnham K.J., Huang X., Voltakis I., Beyreuther K., Masters C.L., Cherny R.A., Bush A.I., Cappai R. (2004). Iron inhibits neurotoxicity induced by trace copper and biological reductants. J. Biol. Inorg. Chem..

[66] Naletova I., Satriano C., Curci A., Margiotta N., Natile G., Arena G., La Mendola D., Nicoletti V.G., Rizzarelli E. (2018). Cytotoxic phenanthroline derivatives alter metallostasis and redox homeostasis in neuroblastoma cells. Oncotarget.

[67] Reichardt L.F. (2006). Neurotrophin-regulated signaling pathways. Philos Trans. R Soc. Lond. B Biol. Sci..

[68] Van der Heide L.P., Ramakers G.M., Smidt M.P. (2006). Insulin signaling in the central nervous system: Learning to survive. Prog. Neurobiol..

[69] Sweatt J.D. (2004). Mitogen-activated protein kinases in synaptic plasticity and memory. Curr. Opin. Neurobiol..

[70] Impey S., McCorkle S.R., Cha-Molstad H., Dwyer J.M., Yochum G.S., Boss J.M., McWeeney S., Dunn J.J., Mandel G., Goodman R.H. (2004). Defining the CREB regulon: A genome-wide analysis of transcription factor regulatory regions. Cell.

[71] Barco A., Bailey C.H., Kandel E.R. (2006). Common molecular mechanisms in explicit and implicit memory. J. Neurochem..

[72] Abel T., Nguyen P.V. (2008). Regulation of hippocampus-dependent memory by cyclic AMP-dependent protein kinase. Prog. Brain Res..

[73] Kowianski P., Lietzau G., Czuba E., Waskow M., Steliga A., Morys J. (2018). BDNF: A Key Factor with Multipotent Impact on Brain Signaling and Synaptic Plasticity. Cell Mol. Neurobiol..

[74] Motamedi S., Karimi I., Jafari F. (2017). The interrelationship of metabolic syndrome and neurodegenerative diseases with focus on brain-derived neurotrophic factor (BDNF): Kill two birds with one stone. Metab. Brain Dis..

[75] Tonks N.K. (2013). Protein tyrosine phosphatases--from housekeeping enzymes to master regulators of signal transduction. FEBS J..

[76] Singh K.B., Maret W. (2017). The interactions of metal cations and oxyanions with protein tyrosine phosphatase 1B. Biometals.

[77] Trusso Sfrazzetto G., Satriano C., Tomaselli G.A., Rizzarelli E. (2016). Synthetic fluorescent probes to map metallostasis and intracellular fate of zinc and copper. Coord. Chem. Rev..

[78] Cheng Q., Song S.H., Augustine G.J. (2017). Calcium-Dependent and Synapsin-Dependent Pathways for the Presynaptic Actions of BDNF. Front. Cell Neurosci..

[79] Crouch P.J., Hung L.W., Adlard P.A., Cortes M., Lal V., Filiz G., Perez K.A., Nurjono M., Caragounis A., Du T. (2009). Increasing Cu bioavailability inhibits Abeta oligomers and tau phosphorylation. Proc. Natl. Acad. Sci. USA.

[80] White A.R., Du T., Laughton K.M., Volitakis I., Sharples R.A., Xilinas M.E., Hoke D.E., Holsinger R.M., Evin G., Cherny R.A. (2006). Degradation of the Alzheimer disease amyloid beta-peptide by metal-dependent up-regulation of metalloprotease activity. J. Biol. Chem..

[81] Wu W., Samet J.M., Silbajoris R., Dailey L.A., Sheppard D., Bromberg P.A., Graves L.M. (2004). Heparin-binding epidermal growth factor cleavage mediates zinc-induced epidermal growth factor receptor phosphorylation. Am. J. Respir. Cell Mol. Biol..

[82] De Souza A.P., Gerlach R.F., Line S.R. (2000). Inhibition of human gingival gelatinases (MMP-2 and MMP-9) by metal salts. Dent. Mater..

[83] Hwang J.J., Park M.H., Koh J.Y. (2007). Copper activates TrkB in cortical neurons in a metalloproteinase-dependent manner. J. Neurosci. Res..

[84] Minami A., Takeda A., Yamaide R., Oku N. (2002). Relationship between zinc and neurotransmitters released into the amygdalar extracellular space. Brain Res..

[85] Hopt A., Korte S., Fink H., Panne U., Niessner R., Jahn R., Kretzschmar H., Herms J. (2003). Methods for studying synaptosomal copper release. J. Neurosci. Methods.

[86] Kubiatowski T., Jang T., Lachyankar M.B., Salmonsen R., Nabi R.R., Quesenberry P.J., Litofsky N.S., Ross A.H., Recht L.D. (2001). Association of increased phosphatidylinositol 3-kinase signaling with increased invasiveness and gelatinase activity in malignant gliomas. J. Neurosurg..

[87] Matsumoto K., Minamitani T., Orba Y., Sato M., Sawa H., Ariga H. (2004). Induction of matrix metalloproteinase-2 by tenascin-X deficiency is mediated through the c-Jun N-terminal kinase and protein tyrosine kinase phosphorylation pathway. Exp. Cell Res..

[88] Iulita M.F., Bistue Millon M.B., Pentz R., Aguilar L.F., Do Carmo S., Allard S., Michalski B., Wilson E.N., Ducatenzeiler A., Bruno M.A. (2017). Differential deregulation of NGF and BDNF neurotrophins in a transgenic rat model of Alzheimer’s disease. Neurobiol. Dis..

[89] Burns A., Iliffe S. (2009). Alzheimer’s disease. BMJ.

[90] Francis B.M., Kim J., Barakat M.E., Fraenkl S., Yucel Y.H., Peng S., Michalski B., Fahnestock M., McLaurin J., Mount H.T. (2012). Object recognition memory and BDNF expression are reduced in young TgCRND8 mice. Neurobiol. Aging.

[91] Hock C., Heese K., Hulette C., Rosenberg C., Otten U. (2000). Region-specific neurotrophin imbalances in Alzheimer disease: Decreased levels of brain-derived neurotrophic factor and increased levels of nerve growth factor in hippocampus and cortical areas. Arch. Neurol..

[92] Connor B., Young D., Yan Q., Faull R.L., Synek B., Dragunow M. (1997). Brain-derived neurotrophic factor is reduced in Alzheimer’s disease. Mol. Brain Res..

[93] Choi S.H., Bylykbashi E., Chatila Z.K., Lee S.W., Pulli B., Clemenson G.D., Kim E., Rompala A., Oram M.K., Asselin C. (2018). Combined adult neurogenesis and BDNF mimic exercise effects on cognition in an Alzheimer’s mouse model. Science.

