# Supplementary material for: The Copper(II)-Assisted Connection between NGF and BDNF by Means of Nerve Growth Factor-Mimicking Short Peptides
Source: Cells. 2019 Apr 1;8(4):301. doi: 10.3390/cells8040301 (PMC6523629; doi:10.3390/cells8040301)
Supplement: Supplementary file 1 [file cells-08-00301-s001.pdf]

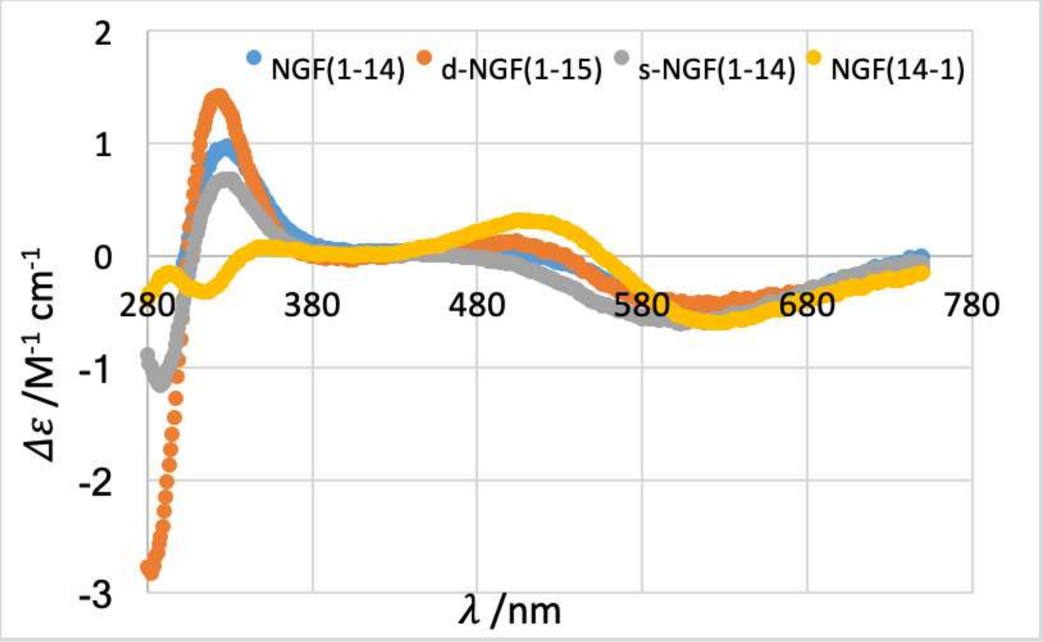

Figure S1. UV-vis CD spectra of copper complexes of NGF(1-14) peptides (1:1 metal to peptide molar ratio, 10^{-3} M, pH=7.4).

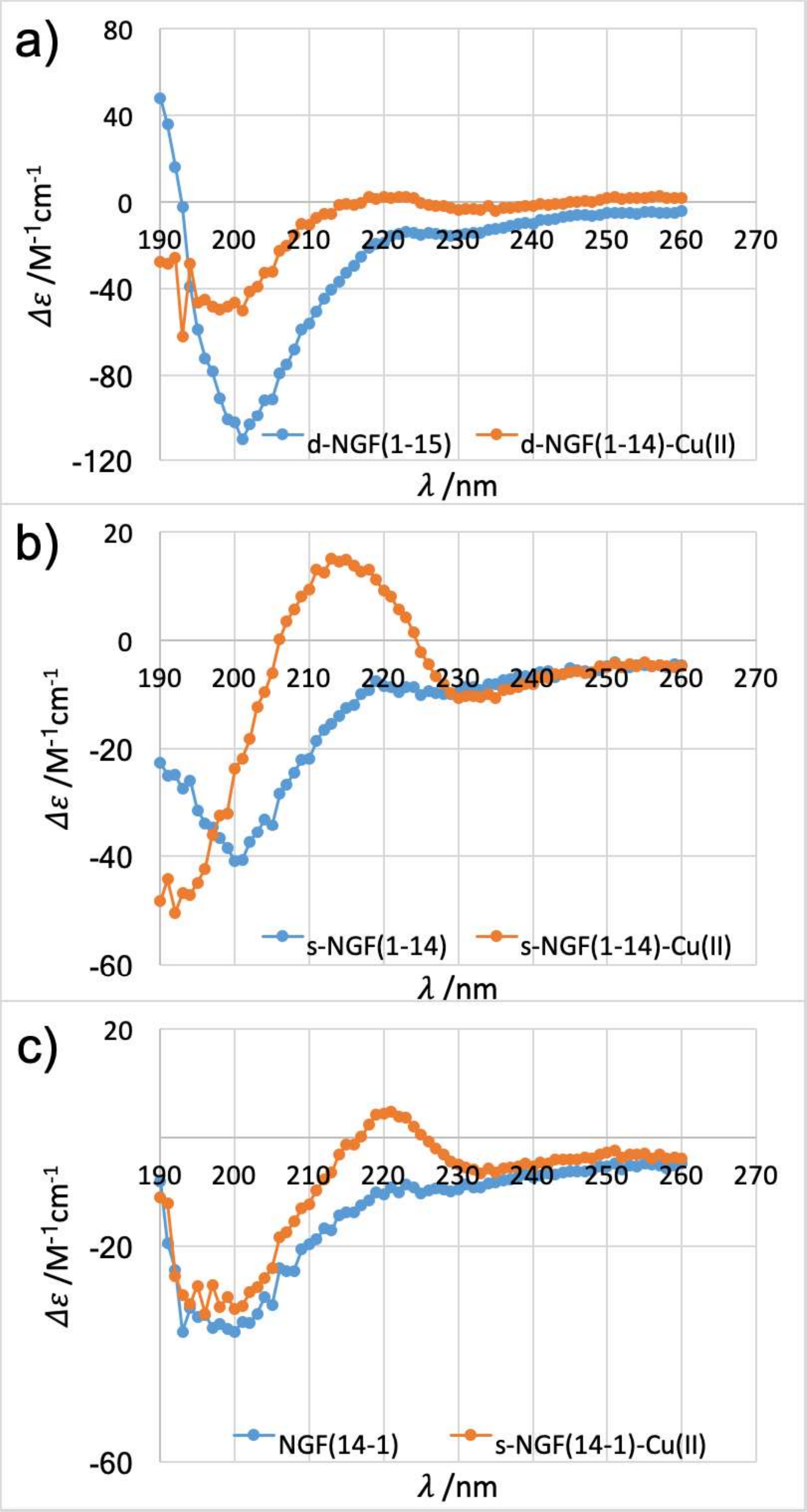

Figure S2. Far UV-CD spectra of NGF(1-14) peptides and their copper complexes (1:1 metal to peptide molar ratio, 10^{-5} M, pH=7.4): a) d-NGF(1-15); b) s-NGF(1-14); c) NGF(14-1).

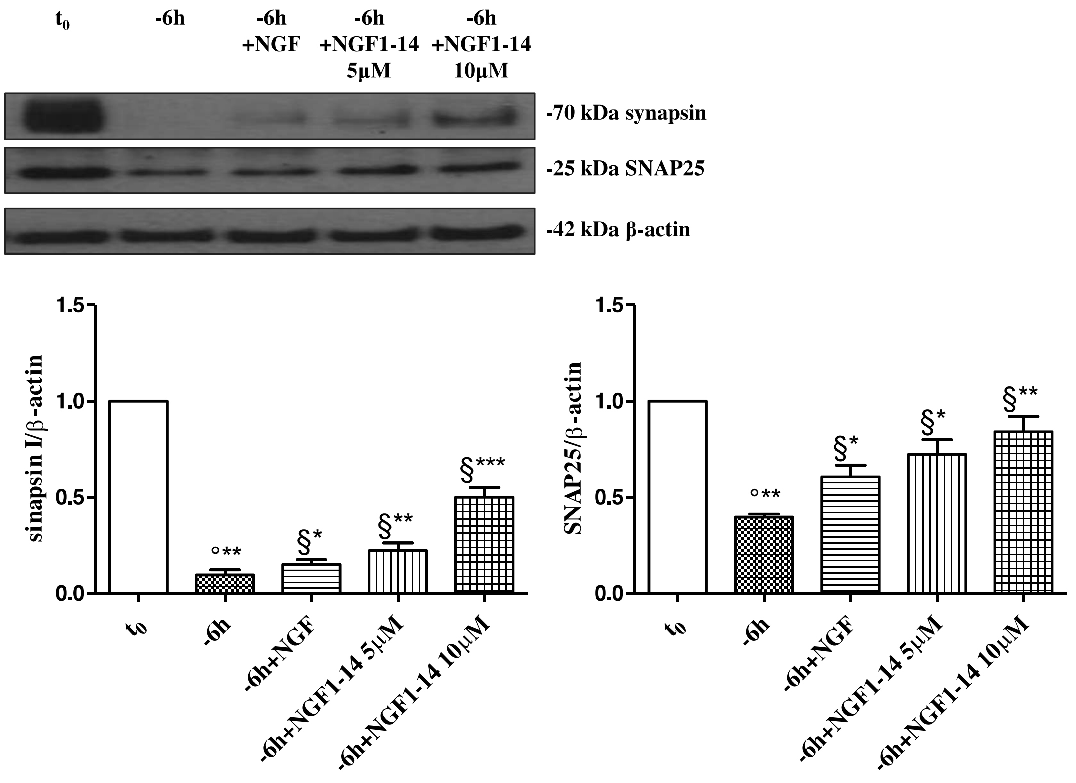

Figure S3. NGF 1-14 is endowed with the same functional phenotype of full-length NGF by blocking the "dying-back" presynaptic degeneration in cholinergic septo-hippocampal neurons. Septal cholinergic-enriched cultures grown chronically in 0.2% B27 media in the presence of exogenous NGF (100ng/ml) from plating were deprived of their trophic support for -6h. At this time point, cells were either harvested (-6h) or re-exposed to NGF (100ng/ml), as reported in (Latina et al., 2017), and to NGF 1-14 and further kept up to 24h when they were eventually collected (-6h+NGF; 6h+NGF 1-14). Equal amounts of total protein extract (40-80μg) were resolved on SDS-PAGE gels and immunoblots were probed with antibodies directed against relevant presynaptic proteins (A)- including synapsin I and synaptosomal-associated protein 25 (SNAP-25). Cropped representative WB are shown. Molecular weights are indicated on the right of the blots and expressed in kDa. Densitometric quantification of immunoreactivity levels (B) was calculated by normalizing the values on the intensity of β-actin, as loading control for each sample/lane. Values are mean ± SEM of at least nine independent experiments and are expressed with respect to B27 0.2% control neurons at t=0 (t0) (°) and at t=--6h of NGF deprivation (§). Statistically significant differences were calculated by unpaired-two tailed t-Student's test (*$p<0.05$, **$p<0.01$ and *** $p<0.0001$).
